# Plastics Derived Endocrine Disruptors (BPA, DEHP and DBP) Induce Epigenetic Transgenerational Inheritance of Obesity, Reproductive Disease and Sperm Epimutations

**DOI:** 10.1371/journal.pone.0055387

**Published:** 2013-01-24

**Authors:** Mohan Manikkam, Rebecca Tracey, Carlos Guerrero-Bosagna, Michael K. Skinner

**Affiliations:** Center for Reproductive Biology, School of Biological Sciences, Washington State University, Pullman, Washington, United States of America; Massachusetts General Hospital, United States of America

## Abstract

Environmental compounds are known to promote epigenetic transgenerational inheritance of adult onset disease in subsequent generations (F1–F3) following ancestral exposure during fetal gonadal sex determination. The current study was designed to determine if a mixture of plastic derived endocrine disruptor compounds bisphenol-A (BPA), bis(2-ethylhexyl)phthalate (DEHP) and dibutyl phthalate (DBP) at two different doses promoted epigenetic transgenerational inheritance of adult onset disease and associated DNA methylation epimutations in sperm. Gestating F0 generation females were exposed to either the “plastics” or “lower dose plastics” mixture during embryonic days 8 to 14 of gonadal sex determination and the incidence of adult onset disease was evaluated in F1 and F3 generation rats. There were significant increases in the incidence of total disease/abnormalities in F1 and F3 generation male and female animals from plastics lineages. Pubertal abnormalities, testis disease, obesity, and ovarian disease (primary ovarian insufficiency and polycystic ovaries) were increased in the F3 generation animals. Kidney and prostate disease were only observed in the direct fetally exposed F1 generation plastic lineage animals. Analysis of the plastics lineage F3 generation sperm epigenome previously identified 197 differential DNA methylation regions (DMR) in gene promoters, termed epimutations. A number of these transgenerational DMR form a unique direct connection gene network and have previously been shown to correlate with the pathologies identified. Observations demonstrate that a mixture of plastic derived compounds, BPA and phthalates, can promote epigenetic transgenerational inheritance of adult onset disease. The sperm DMR provide potential epigenetic biomarkers for transgenerational disease and/or ancestral environmental exposures.

## Introduction

Epigenetic transgenerational inheritance involves the transmission of a phenotypic alteration to subsequent generations (F3) through germline epimutations following ancestral environmental exposure of a gestating F0 generation female [1], [2]. Previous studies [2], [3], [4] with the agricultural fungicide vinclozolin administered to gestating rats and mice during the gonadal sex determination period promotes a male germline epigenome reprogramming to induce transgenerational adult-onset disease. This modification of germline epigenetic programming occurs during the gonadal sex determination period when the germline DNA is demethylated and remethylated in a sex specific manner [1], [5]. This modified epigenetic programming of the male germline subsequently leads to all tissues propagated from this sperm to have differentially altered epigenomes and transcriptomes that can influence development of adult-onset disease. The altered epigenome in the germline is transmitted through subsequent generations due to apparent permanent imprinted-like DNA methylation properties [4]. These germline mediated epimutations enable epigenetic transgenerational inheritance of altered phenotypes.

Environmental chemicals such as vinclozolin and the pesticide methoxychlor [2] are known to promote epigenetic transgenerational inheritance of adult-onset diseases. The current study was designed to investigate the actions of a mixture of plastic derived endocrine disruptor compounds bisphenol-A (BPA), bis(2-ethylhexyl)phthalate (DEHP) and dibutyl phthalate (DBP). This mixture of plastic derived compounds was selected due to the common exposures in human populations such as military personnel [6]. Bisphenol-A is used to make polycarbonate plastic and epoxy resins which are in turn used in a variety of plastic items such as water bottles, sports equipment, medical and dental devices, dental fillings and sealants, household electronics and eyeglass lenses [7]. Bisphenol A is an endocrine disruptor with widespread exposure and multiple effects including impaired reproductive capacity, promotion of obesity and metabolic disease [8], [9], [10], [11], [12]. DEHP is widely used as a plasticizer in manufacturing of articles made of polyvinyl compounds [13] and it is considered a reproductive and developmental toxicant in humans and animals [14]. DBP is a phthalate used primarily as plasticizer to add flexibility to plastics. DBP is used as a component in latex adhesives, cosmetics, in cellulose plastics, and as a solvent for dyes. Exposure of pregnant females to high doses of DBP (greater than 500,000 µg/kg BW/day) causes reduced fetal survival, reduced birth weights among surviving offspring, skeletal malformations and reproductive abnormalities in both male and female offspring associated with reduced fertility [15]. These three endocrine disruptors (BPA, DEHP and DBP) have been shown to be derived from various plastic bottles [16] and are common exposures in humans [6], [17].

Previous studies with bisphenol-A or phthalates have primarily focused on F0 or F1 generation studies [18]. Actions on the F0 and F1 generations involve direct effect of the exposure on the gestating female or fetus, so is a multigenerational exposure [1]. Exposure of an F0 generation gestating female also exposes the germline in the F1 generation fetus that will develop into the F2 generation. The F3 generation is required to eliminate the possibility of direct exposure effects [1]. The current study focused on transgenerational effects and analyzed F3 generation in comparison with the direct exposure F1 generation. There has been only one study that documented transgenerational effects of bisphenol-A for three generations involving testis abnormalities [19]. The current study used doses of a <1% fraction of the oral LD50 dose for bisphenol-A or phthalates DEHP and DBP through intraperitoneal injection. Previous studies have suggested these doses do not produce overt toxicity (changes in litter size, sex ratio, or mean weights) in the F1 generation [20]. The doses selected are considered low for previous rodent exposures [11], [21], [22], [23], [24], [25], [26], [27], [28], [29], [30], but are high in relation to common human exposures. Therefore, the study was designed to examine the potential pharmacological actions of the compounds to influence epigenetic transgenerational inheritance and not designed to do risk assessment analysis. The observations of the current study can now be used to more effectively design risk assessment studies.

The current study examined the hypothesis that the exposure of a gestating female during the fetal gonadal sex determination period to the plastics mixture (BPA, DEHP and DBP) promotes epigenetic transgenerational inheritance of adult onset disease. In the present study diseases of the testis, prostate, kidney, ovary, tumor development, and obesity were evaluated in 1-year old rats of F1 and F3 generations. Phenotypes observed in the F1 generation animals are induced by a direct chemical exposure of the fetus and somatic cells. However, effects observed in the F3 generation animals are due to epigenetic transgenerational inheritance through the germline and not due to any direct effect of the chemical exposure [31]. Therefore, phenotypes or diseases observed in F1 and F3 generation animals are not due to the same mechanism and are often distinct. This study documents epigenetic transgenerational inheritance of testis and ovary diseases, pubertal abnormalities, and obesity in F3 generation offspring after the gestating ancestors (great-grandmothers) were exposed to a mixture of plastic derived compounds. This study further documents the ability of these environmental exposures to induce epigenetic transgenerational inheritance of sperm epimutations.

A recent study compared the actions of the plastic compound mixture (BPA, DEHP, DBP) with a pesticide mixture, dioxin and a hydrocarbon mixture on postnatal day 120 (P120) rats which demonstrated all exposures induced F3 generation reproductive abnormalities [20]. Observations demonstrated similar transgenerational disease phenotypes, but unique transgenerational sperm epimutations [20]. The majority of adult onset disease develops later in life (>6 mo age in rat) [32] and is not present at P120 in rats. The current study extends these previous observations [20] to examine the plastic compound mixture's actions on F3 generation animals. In addition, the transgenerational sperm epimutations previously identified are more thoroughly investigated.

## Results

### Transgenerational Adult-Onset Disease Analysis

The experimental design included exposure of outbred Harlan Sprague Dawley gestating female rats to daily intraperitoneal injections of DMSO vehicle (control) or a mixture of plastic derived compounds (BPA, DEHP and DBP), designated as “plastics” and “lower dose plastics” (one half dose as plastics group) during fetal days 8 to 14 of gestation. The F1 generation rat offspring born to different exposed females were bred to obtain the F2 generation. The F3 generation animals were obtained by breeding non-littermate females and males of the F2 generation. No sibling or cousin breeding was used to avoid any inbreeding artifacts in generating the different lineages. Randomly selected offspring from different litters of the F1 and F3 generations were aged to one year and euthanized. Body and organ weights were measured and examined for disease/abnormalities. The testis, prostate, kidney and ovary were examined for histopathology as outlined in Methods.

Potential overt toxicity from direct fetal exposure to plastics or lower dose plastics in the F1 generation animals was determined and comparisons were made to the F3 generation animals through analysis of body weight and organ weights (Table S1A). Both ovarian and uterine weights decreased in the F1 generation rats of lower dose plastics lineage compared to control lineage. Only uterine weights decreased in the F3 generation rats of plastics and lower dose plastics lineage compared to control lineage. There were no effects on body weight and weights of the testis, prostate, seminal vesicle, epididymis and kidney of 1-year old male F1 generation rats. The seminal vesicle weights of the F3 generation plastics and lower dose plastics lineage decreased compared to control lineage. There was also a decline in epididymal weight in lower dose plastics lineage (Table S1B). Hormone concentrations were measured in the F3 generation control, plastics and lower dose plastics lineages to assess any endocrine alteration in serum sex steroid levels. Serum testosterone concentrations in the 1-year-old F3 generation male rats from plastics or lower dose plastics lineages did not differ from those of control lineage. Serum estradiol concentrations in female rats during proestrus-estrus phase or diestrus phase were also not altered in plastics and lower dose plastics lineages compared to control lineage (Figure S2). No statistical difference (p>0.05) was observed in litter size (average 12) or sex ratio (50∶50) in the F1 or F3 generation control versus plastics lineages. These combined observations suggest there is no major endocrine or overt toxicity from the plastics or lower dose plastics exposures at the doses administered.

**Figure 1:**
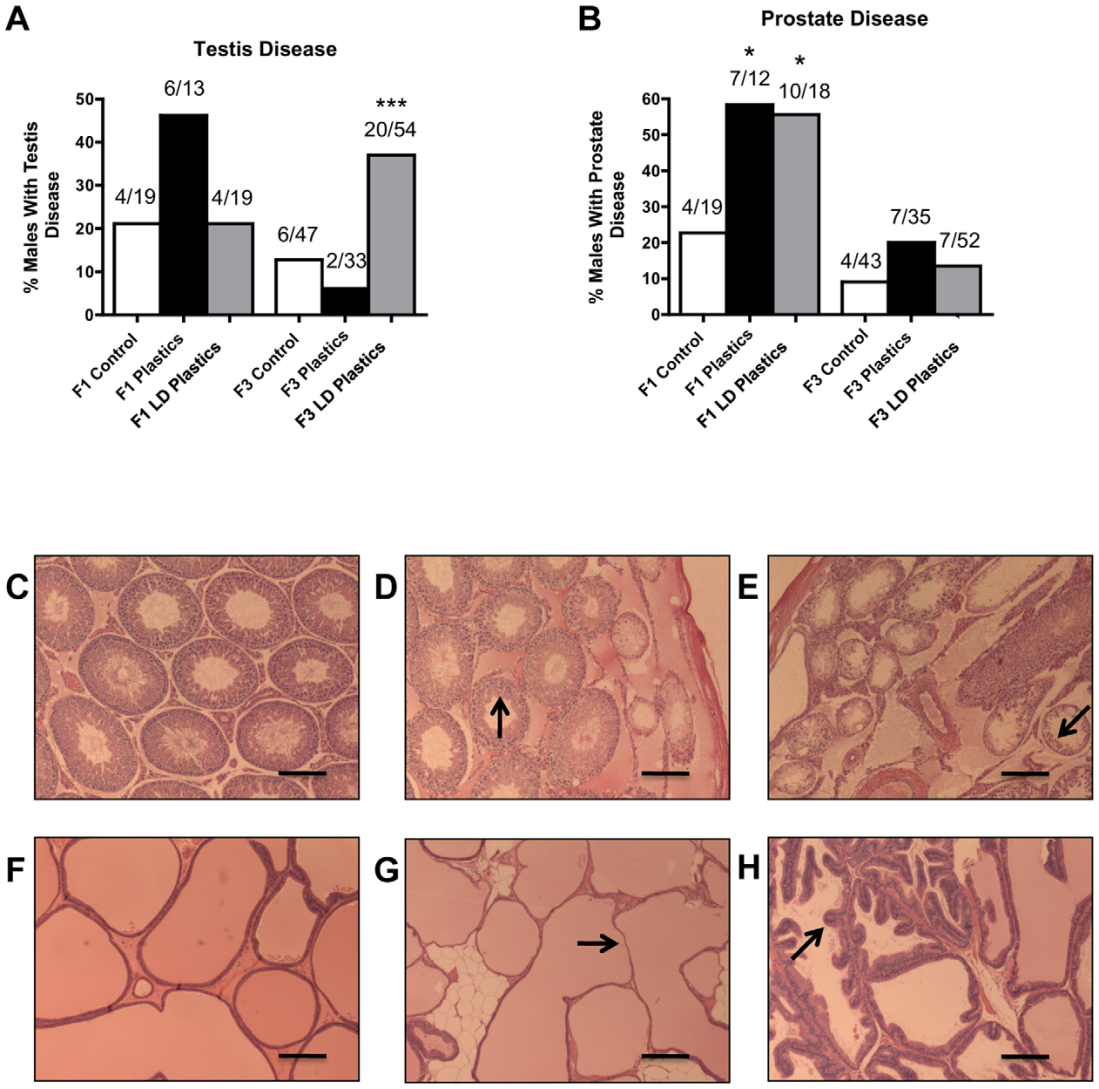
Adult-onset testis disease and prostate disease in males from control, plastics and lower dose (**LD**) **plastics** (**BPA, DEHP and DBP**) **lineages.** Percentages of males with testis (panel A) or prostate disease (panel B) in F1 and F3 generations are presented. The actual number of diseased rats/total number of rats in each group are shown above the respective bar graphs (* P<0.05; *** P<0.001). Representative micrographs (Scale bar  = 200 μm) showing histopathology images of adult-onset transgenerational testis and prostate disease in plastics (panels D, and G) and lower dose (LD) plastics lineages (panels E and H) compared to F3 control lineage (panels C and F). Testis sections from F3 generation animals in plastics and lower dose (LD) plastics lineages showed histopathology including azoospermic and atretic seminiferous tubules, presence of vacuoles in basal regions of seminiferous tubules, sloughed cells in center of seminiferous tubule and lack of seminiferous tubule lumen (arrows). Prostate sections showed epithelial atrophy and hyperplastic ductular epithelium (arrows).

**Figure 2:**
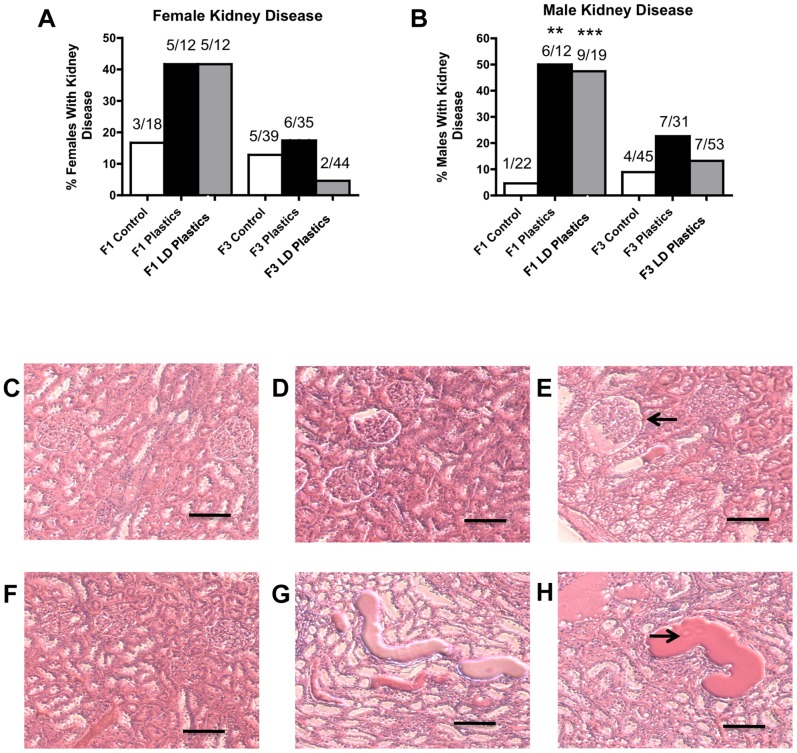
Adult-onset kidney disease in males or females from control, plastics and lower dose (**LD**) **plastics** (**BPA, DEHP and DBP**) **lineages.** Percentages of females (panel A) and males (panel B) with kidney disease in F1 and F3 generations are presented. The actual number of diseased rats/total number of rats in each group are shown above the respective bar graphs (** P<0.01; *** P<0.001). Representative micrographs (Scale bar  = 200 μm) showing histopathology images of adult-onset transgenerational kidney disease in F3 generation plastics (panels D and G) and lower dose (LD) plastics lineages (panels E and H) compared to F3 control lineage (panels C and F). Kidney sections showed Bowman's capsule abnormality and proteinaceous fluid filled cysts (arrows).

**Figure 3:**
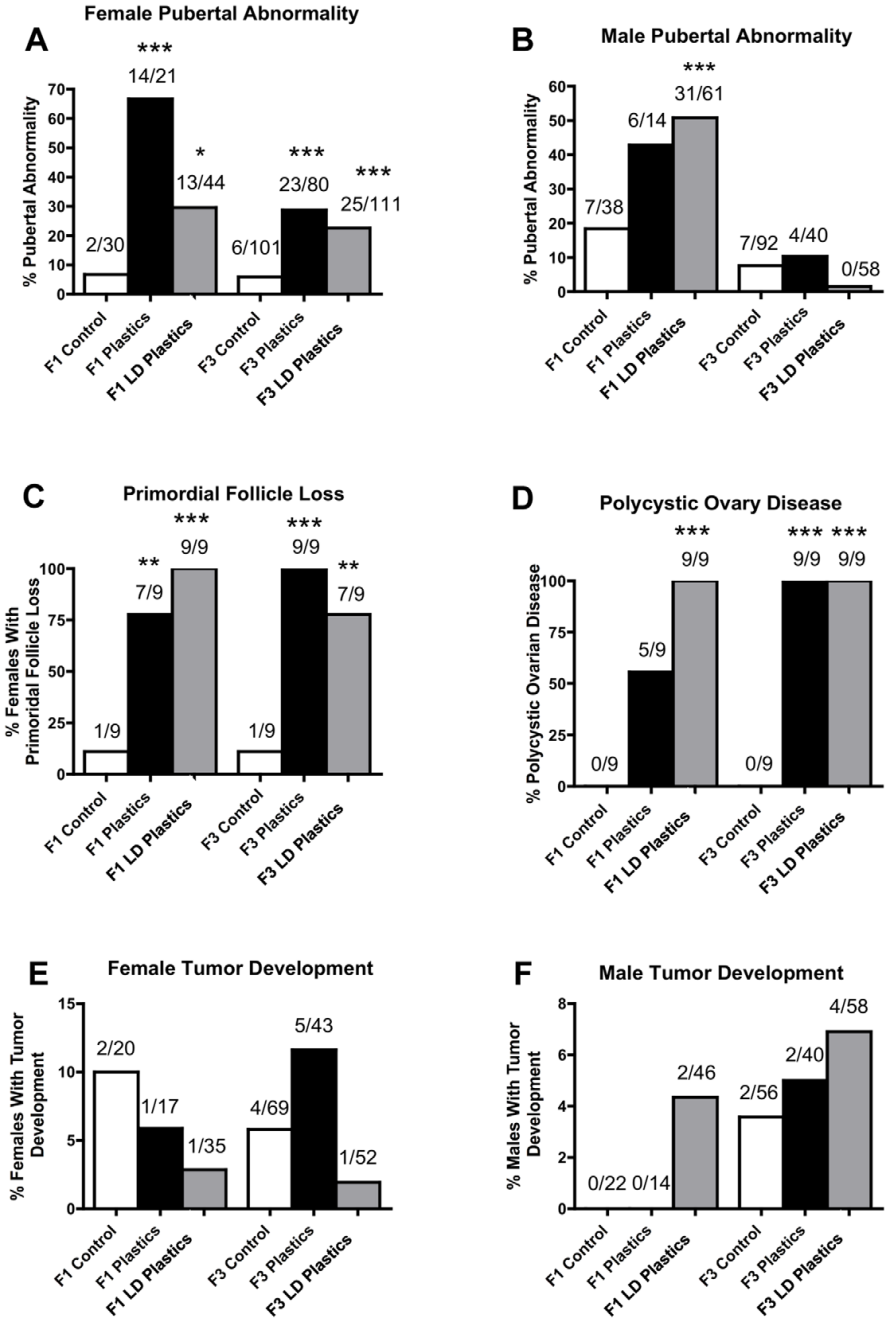
Pubertal abnormalities, primordial follicle loss, polycystic ovary disease and tumor development from control, plastics, or lower dose (**LD**) **plastics** (**BPA, DEHP and DBP**) **lineages.** Percentages of females (panel A) and males (panel B) with pubertal abnormalities, or those females with primordial follicle loss (panel C) or polycystic ovary disease (panel D), and tumor development in females (panel E) and males (panel F) in F1 and F3 generations are presented. The actual number of diseased rats/total number of rats in each group are shown above the respective bar graphs (* P<0.05; ** P<0.01; *** P<0.001).

**Figure 4:**
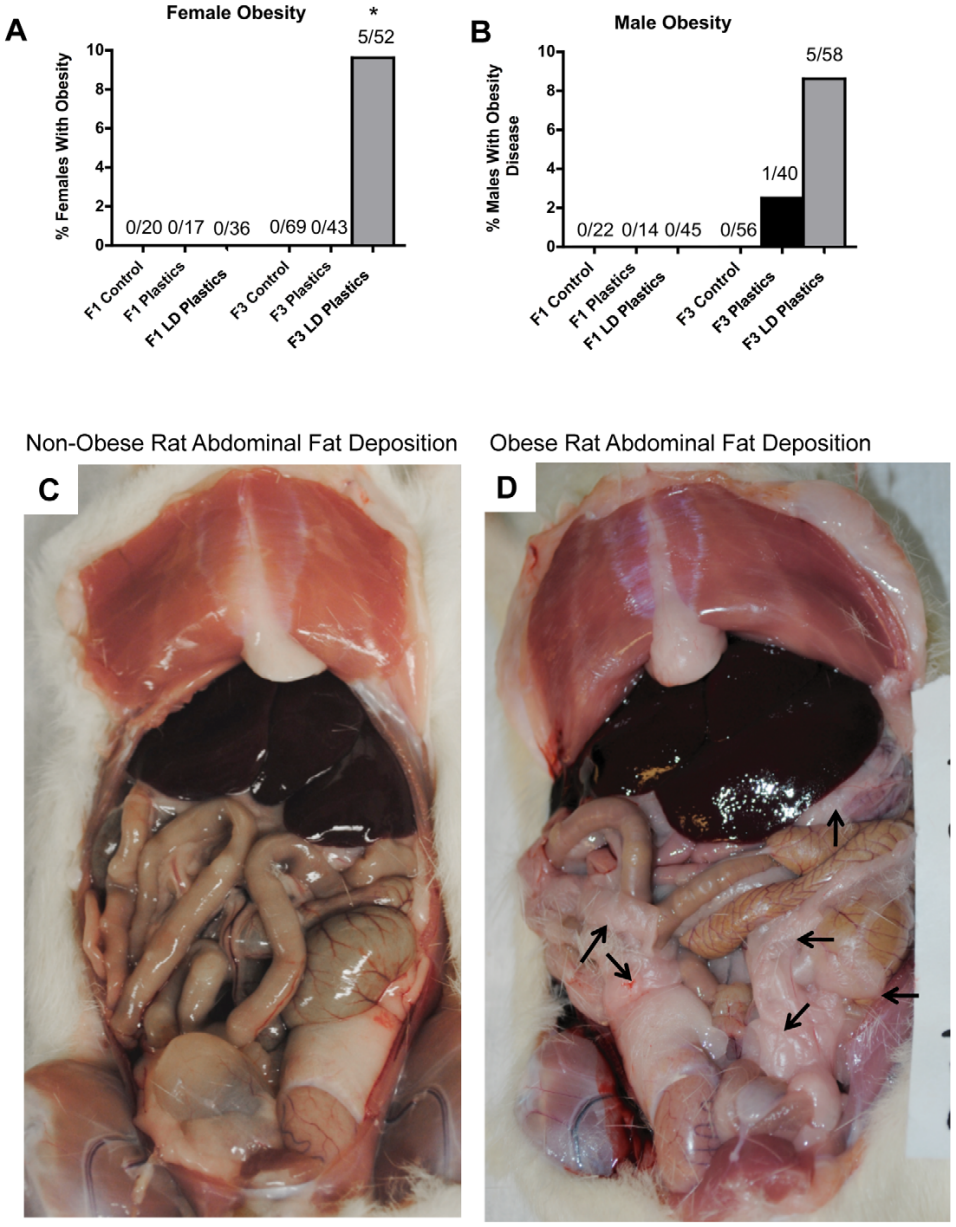
Obesity developed in control, plastics, or lower dose (**LD**) **plastics** (**BPA, DEHP and DBP**) **lineages.** Percentages of females (panel A) and males (panel B) with obesity in F1 and F3 generations are presented. The actual number of diseased rats/total number of rats in each group are shown above the respective bar graphs (* P<0.05). Abdominal fat deposition in F3 generation 1yr old rats from non-obese (C) and obese (D) animals. Pink colored fat deposition over most organs noted in panel B (arrows).

One of the major disease/abnormality phenotypes observed in the F1 and F3 generation males of plastics lineage was testicular disease. There was a significant increase in the incidence of transgenerational testis disease in the F3 generation males of lower dose plastics lineage (Figure 1A). Testis histopathological abnormalities include the azoospermic and atretic seminiferous tubules, the presence of vacuoles in basal regions of the seminiferous tubules, the sloughed spermatogenic cells in the center of seminiferous tubules and the lack of seminiferous tubule lumen (Figures 1D, and 1E). Further analysis of testis abnormalities determined the number of apoptotic spermatogenic cells within the testis of male rats in plastics and lower dose plastic lineages. Significantly higher spermatogenic cell apoptosis in males of the F3 generation lower dose plastics lineage was observed (Figure S1). Interestingly, reduced germ cell apoptosis was observed in males of the F1 generation plastics and lower dose plastics lineages and of the F3 generation plastics lineage. Therefore spermatogenic defects that were previously observed in vinclozolin lineage F3 generation rats (2) were also present in the F3 generation rats from the lower dose plastics lineage. The F1 generation males of lower dose plastics lineage had increased incidence of prostate disease/abnormalities. The prostate histopathological abnormalities included hyperplastic or atretic ductular epithelium (Figure 1B). Likewise, the F1 generation males of plastics and lower dose plastics lineages showed an increased incidence of kidney disease. The kidney histopathological abnormalities included Bowman's capsule abnormalities and proteinaceous fluid filled cysts (Figure 2B). As previously reported [20] a significant increase in pubertal abnormalities (comprising early or delayed onset of puberty) was documented in the F1 generation male rats of the lower dose plastics lineage compared to the control lineage (Figure 3B). The incidences of pubertal abnormalities in the F1 generation males from plastics or lower dose plastics lineages were 43% and 51% respectively, with the majority of these having a delayed pubertal onset. The incidence of pubertal abnormalities in the F1 generation males of the control lineage was only 18% with the majority of these animals having a delayed pubertal onset. No changes were observed in the incidence of pubertal abnormalities in the F3 generation males from plastics or lower dose plastics lineages when compared to the control F3 generation lineage males (Figure 3B). The F3 generation males from plastics lineage did not show any significant change in transgenerational prostate disease (Figure 1B) or kidney disease (Figure 2B). Tumor development in the F1 and F3 generation males was also investigated and no significant difference in tumor development between the control and plastics lineages were observed (Figure 3F). The predominant tumor observed were mammary gland tumors, with some isolated tumors in the skin, spleen, urinary bladder, cerebellum, lung and liver detected. Obesity was assessed with an increase in body weight and dramatic increase in abdominal adiposity, as shown in Figure 4D. No obesity was detected in the male F1 generation control or plastic lineage animals. Obese males were observed in the F3 generation and the body weight of the non-obese (509±32) and obese (555±32) males were statistically different (p<0.008). The abdominal adipose tissue was present on most organs and dramatically increased in the obese animals (Figure 4D) compared to the non-obese animals (Figure 4C). The F3 generation males of lower dose plastics lineage had a tendency to have an increased incidence of obesity (p = 0.0697). Therefore, ancestral exposure to lower dose plastics promoted transgenerational testis disease and obesity, but not prostate or kidney disease, to their unexposed F3 generation male descendants.

There were a greater number of transgenerational diseases in the F3 generation female rats from plastics and lower dose plastics lineages. These included pubertal abnormalities (Figure 3A), and ovarian disease. Ovarian disease involved both primordial follicle loss (Figure 3C), as shown by a severe reduction in the number of primordial follicles per ovary section [20], and polycystic ovarian disease (Figure 3D), as characterized by an increase in the number of cysts. The increase in the proportion of the F3 females of plastics and lower dose plastics lineages with ovarian disease was dramatic. In the control lineage F1 and F3 generation females only one out of 9 had primordial follicle loss. In contrast, the majority of the F1 generation plastics lineage (78%, 7/9), all of the lower dose plastics lineage F1 generation females (100%, 9/9), all of the F3 generation plastics lineage (100%, 9/9), and majority of the lower dose plastics lineage F3 generation females (78%, 7/9) examined had a significant loss of primordial follicles with a reduced ovarian follicular reserve (Figure 3C). This condition is associated with potential future development of primary ovarian insufficiency. In the control lineage none of the females in either the F1 or F3 generations examined developed polycystic ovaries. In contrast, the majority of the F1 generation plastics lineage (56%, 5/9), all of the lower dose plastics lineage F1 generation females (100%, 9/9), and all of the plastics and lower dose plastics lineage F3 generation females (100%, 9/9) examined had a significant increase in number of cysts within the ovary (Figure 3D). Polycystic ovarian disease is the most common ovarian disease in women of reproductive age. Therefore, the observations demonstrate epigenetic transgenerational inheritance of ovarian disease following ancestral exposure to the plastic compounds.

The incidences of pubertal abnormalities in the F1 generation females of plastics and lower dose plastics lineages were 67% and 30% respectively with the majority of these animals having delayed pubertal onset (Figure 3A). The incidences of pubertal abnormalities in the F3 generation females of plastics and lower dose lineages were 29% and 23% respectively with the majority of these animals having an early onset of puberty. The F1 and F3 generation females from plastics or lower dose plastics lineages did not have an increased incidence of adult onset kidney disease (Figure 2A) or tumor development (Figure 4A). The tumors observed were primarily mammary gland tumors, with skin and small intestine isolated tumors also indicated. The lower dose plastics lineage F3 generation females did have a significant increase in obesity (Figure 4C). The obesity phenotype involved an increase in female body weight (332±10 obese and 283±48 non-obese) and significant increase in abdominal fat deposition and adiposity of most organs, Figure 4D. These combined observations indicate ancestral exposure to plastics and low dose plastics promotes transgenerational inheritance of pubertal abnormalities, ovarian disease and obesity in the F3 generation female descendants.

Other less frequent diseases were observed in the plastics and low dose plastics lineages. These included constipation, swollen intestinal lymph nodes, small seminal vesicles, sinus histiocytosis and stomach abnormalities in the F1 generation animals of the plastic lineage. Small seminal vesicles may be a developmental defect. Histiocytosis and swelling of intestinal lymph nodes and stomach abnormality are related to inflammatory processes. Low frequency diseases in the F3 generation animals of plastic lineage included blindness, cataract of the eye, focal fat necrosis, histiocytosis, interstitial pneumonia, liver degeneration, sinusitis, seizures and tremors. The F3 generation animals from the lower dose plastics lineage also developed unique low frequency diseases/abnormalities including liver disease (cirrhosis), swollen epididymis and vulvar abscess. Focal fat necrosis is usually a sign of inflammation due to contusions or constant sitting. Lack of activity from constant sitting may predispose the rat to obesity as well. Histiocytosis, pneumonia, sinusitis, swollen epididymis and vulvar abscess indicate inflammatory abnormalities. Seizures and tremors indicate neural dysfunction. Liver degeneration and cataract may indicate premature aging. Blindness may be due to retinopathy or abnormal blood vessel growth in the eye. These various diseases were infrequent but more predominant in animals of the plastics and low dose plastics lineages.

**Figure 5:**
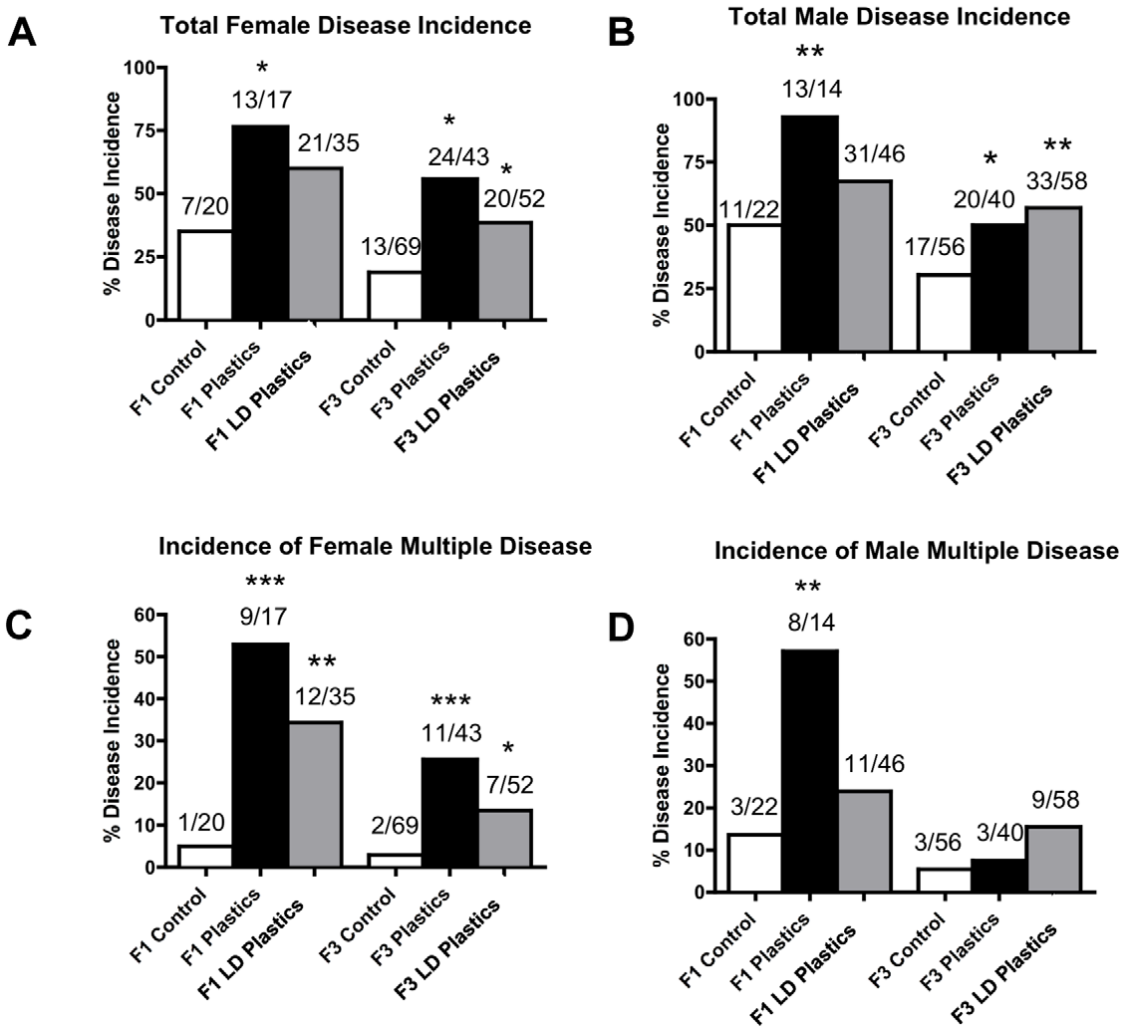
Adult-onset disease/abnormalities in rats from control, plastics, or lower dose (**LD**) **plastics** (**BPA, DEHP and DBP**) **lineages.** Incidences of total female disease (panel A), total male disease (panel B), female multiple disease (panel C) and male multiple disease (panel D) in F1 and F3 generations are presented. The actual number of diseased rats/total number of rats in each group are shown above the respective bar graphs (* P<0.05; ** P<0.01; *** P<0.001).

The incidence of disease/abnormality in individual rats in control, plastics and low dose plastics lineages is presented in Tables S2A (F1 generation females), Table S2B (F1 generation males), Table S3A (F3 generation females) and Table S3B (F3 generation males). These tables list the occurrence of diseases for each rat and clarify the number of animals for each specific disease/abnormality assessment. The incidence of total disease/abnormality increased significantly in the F3 generation females of both plastics and lower dose plastics lineages (Figure 5A). The incidence of total disease/abnormality increased in the F1 generation females of plastics lineage only (Figure 5A). The incidence of multiple disease/abnormalities increased significantly in the F1 and F3 generation females of plastics and lower dose plastics lineages (Figure 5C). The incidence of total disease/abnormality increased significantly in the F3 generation males of plastics and lower dose plastic lineages (Figure 5B). The F1 generation males of plastics lineage, but not lower dose plastics lineage, showed a significant increase in the incidence of total disease/abnormalities (Figure 5B) and in the incidence of multiple disease/abnormalities. Ancestral exposure to plastics and lower dose plastics increased the overall incidence of transgenerational adult onset diseases in both females and males.

### Transgenerational Effects on the Sperm Epigenome

**Figure 6:**
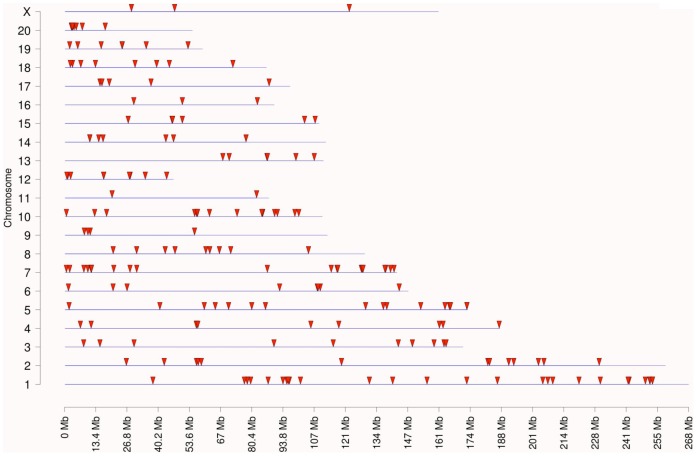
Gene network analysis for differential DNA methylation regions (**DMR**) **associated genes in the F3 generation plastics lineage sperm.** Chromosomal locations for transgenerational DMR detected with MeDIP-Chip are indicated with arrowheads. The chromosomal size and number are presented. There were 197 DMR in sperm DNA from F3 generation plastics lineage compared to control lineage.

**Figure 7:**
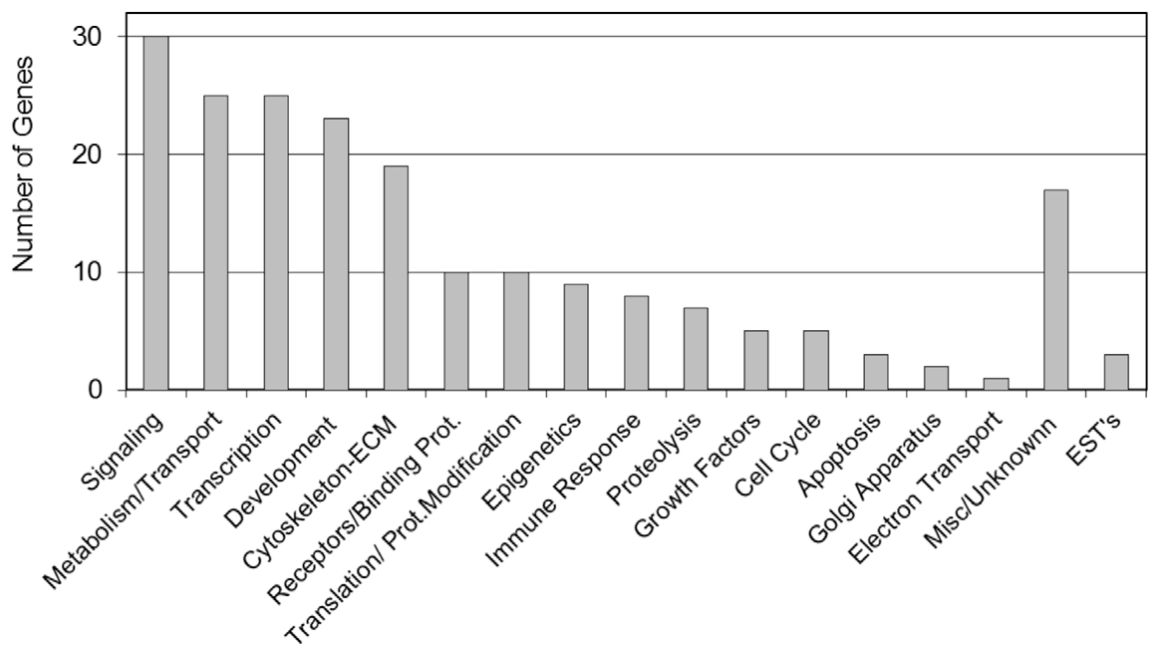
The F3 generation plastics lineage sperm DMR associated gene functional categories. The number of DMR associated genes correlating to a specific gene functional category is presented including those with unknown function and expressed sequence tags (EST).

**Figure 8:**
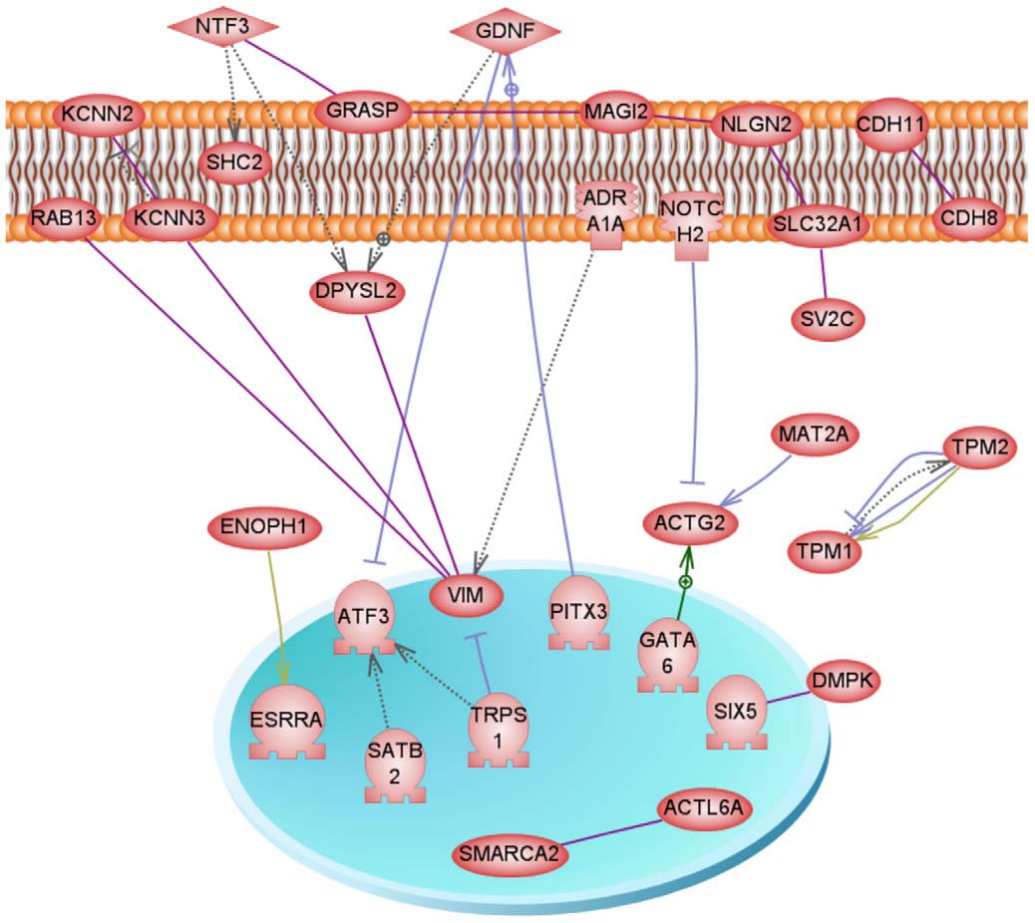
Gene network analysis for differential DNA methylation regions (**DMR**) **associated genes in the F3 generation plastics lineage sperm.** Direct connection (functional or binding) genes are shown according to their location in the cell. Genes not shown are not connected. Node shapes code: oval and circle – protein; diamond – ligand; circle/oval on tripod platform – transcription factor; ice cream cone – receptor. Arrows with plus sign show positive regulation/activation, arrows with minus sign – negative regulation/inhibition; gray arrows represent regulation, lilac – expression, purple – binding, green – promoter binding, and yellow – protein modification.

Environmentally induced epigenetic transgenerational inheritance of adult onset disease involves an altered germline epigenome transmission between generations. The transgenerational F3 generation control and plastics lineage sperm epigenomes were previously analyzed [20] and compared using a methyl cytosine antibody chromatin immunoprecipitation (MeDIP) followed by a genome-wide promoter tiling array chip (MeDIP-Chip) assay [4]. The sperm DNA samples from rats of the F3 generation control and plastics lineages were analyzed and previously reported [20]. Three different experimental pools of F3 generation control and plastics lineage MeDIP were generated and each pool contained sperm DNA from three different animals each from a different litter. A comparative hybridization with the MeDIP-Chip assay was performed as described in the Methods to identify differential DNA methylation regions between the control and plastics lineage sperm pools. This analysis identified statistically significant differential DNA methylation regions (DMR) in 197 different promoters [20] with an average 500 bp in size, Table S4. The DMR previously identified [20] were more thoroughly analyzed in the current study. The gene network described below identified a highly interconnected DMR associated gene that was selected for confirmation with an MeDIP-quantitative (Q) PCR analysis. This DMR associated gene was *Gdnf* and had a 38.1 fold increase (p<0.05) in the plastic lineage MeDIP compared to control using the MeDIP-QPCR analysis. Therefore, the MeDIP-QPCR analysis for this gene confirmed the previously reported MeDIP-Chip analysis [20] for this DMR in postnatal 120 day old males. Future studies are needed to assess the DMR in 1 year old animals. The chromosomal locations of all the differentially methylated regions (DMR) are presented in Figure 6. The sperm DMR (termed epimutations) were present throughout the genome on all chromosomes examined. The functional gene categories of the genes associated with the DMR are shown in Figure 7 and Table S4. Therefore, the exposure to a plastic compound mixture induced a transgenerational sperm epigenome alteration. Analysis of the genes associated with the 197 DMR for potential correlated cellular pathways and processes did not identify pathways with a predominance of DMR associated genes, Table S5. A further analysis was performed to identify a potential direct connection (functional and/or binding connections) gene network associated with the DMR, Figure 8. The network contained a number of extracellular, membrane, cytoplasmic and nuclear localized genes associated with the DMR identified. The glial derived neurotrophic factor (*Gdnf*) and neurotrophin 3 (*Ntf3*) cellular signaling pathways and processes appear to be involved in the gene network identified. Therefore, plastics derived compounds induced a transgenerational alteration in the sperm epigenome and the DMR epimutations potentially influence a gene network of specific cellular pathways.

**Figure 9:**
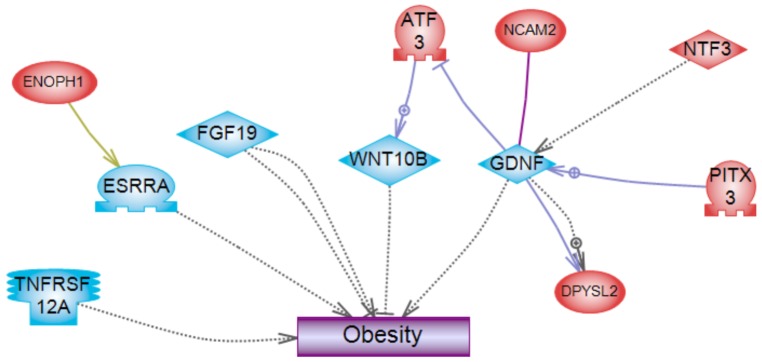
Genes with known links with obesity that correlate with F3 generation plastic lineage sperm DMR associated genes. The correlated DMR associated genes with associations with obesity are presented. The DMR associated genes with indirect connections to the direct connection genes are also presented.

A final analysis of the genes associated with the DMR previously identified [20] examined genes previously shown to be correlated to the pathologies observed. A number (five) of the DMR associated genes correlated to known obesity related genes as shown in Figure 9. Other DMR associated genes (six) had indirect connections through the five direct connection genes *Tnfrsf12a*, *Esrra*, *Fgf19*, *Wnt10b* and *Gdnf*. No genes were found associated with ovarian or testis diseases with direct connections. Interestingly, the *Gdnf* was also observed in the gene network identified, Figure 8. Therefore, previously identified genes that appear to be involved in obesity correlated to a number of the genes associated with the DMR epimutations.

## Discussion

The current study was designed to investigate the actions of a plastic compound mixture to promote epigenetic transgenerational inheritance of adult onset disease. Gestating female rats, designated F0 generation, were exposed to a plastics or lower dose (one-half dose) plastics mixture (BPA, DEHP and DBP) or DMSO vehicle control daily during embryonic days 8–14 of development. The F1 generation progeny were bred to produce the F2 generation, which were bred to obtain the F3 generation. Only the F0 generation females and not the F1, F2 and F3 generation individuals were treated. The F1 and F3 generation animals were aged to one year and then euthanized. Tissues were collected, fixed, sectioned and stained for histopathological examination. Tissues examined included testis, prostate, kidney and ovary. Previously, epididymal sperm were collected from P120 day old males, DNA isolated, and transgenerational F3 generation sperm epigenomes (DNA methylation) were examined with an MeDIP-Chip analysis [20]. The current study more thoroughly investigated these epimutations. The chromosomal locations, a gene network and associated gene functions of the differential methylation regions (DMR) associated genes were identified.

The actions of plastic derived endocrine disruptor compounds have been documented in previous studies primarily using direct exposure studies. The documented actions of bisphenol A (BPA) include altered pubertal onset [33], [34], disruption of estrous cycles [35], [36], prostate disease [37], [38], [39], prostate neoplasia [37], [40], abnormal mammary gland development and presence of intraductal hyperplasia and preneoplastic lesions in adults [41], [42], [43], [44], alterations in the uterus (cystic endometrial hyperplasia) and ovary (cystic ovaries) abnormalities [41], [45]. BPA induced brain and behavioral changes include abnormal development of sexually dimorphic hypothalamic regions [46], [47], [48], [49], abnormal steroid receptor levels [50], [51], [52], aberrant behavior including hyperactivity [53], [54], heightened aggressiveness [55], distorted sociosexual behavior [56], changed cognitive and anxiolytic behaviors [57], and enhanced susceptibility of addiction [54], [58]. BPA alters body weight and body composition [10], [33], [35], [59], [60], [61] and abnormal glucose homeostasis [62]. Therefore, early life BPA direct exposure promotes a variety of adult onset disease states.

Phthalates DEHP and DBP are known reproductive and developmental toxicants [14], [15], [63]. Toxicity of these phthalate esters on male reproductive function include testicular seminiferous tubule atrophy and germ cell degeneration [64], [65], [66], [67], and male reproductive tract abnormalities consistent with androgen dependent development and impaired testicular function [68], [69], [70], [71], [72], [73]. The phenotypic alterations included cryptorchidsm, hypospadias, agenesis of sex accessory organs, testicular injury, reduced daily sperm production, delayed preputial separation, permanent retention of nipples and decreased (feminized) anogenital distance. Fetal exposure effects of DEHP and DBP include reduction in testosterone secretion [74], [75] and increase in the diameter of seminiferous cords and induction of gonocyte multinucleation in rat fetal testis [76]. The female reproductive effects of phthalates include prolonged estrous cycles, reduced serum estradiol levels and absence of ovulation in adult rats [77]. Decrease in fertility [78], [79], disruption of pregnancy [27], abortions, fetal teratogenic abnormalities, skeletal and visceral malformations, delay in the age of pubertal onset [80], [81] and altered number of ovarian follicles [81] are other observed direct exposure effects of phthalates in females.

The current study was designed to examine the actions of pharmacological doses of the plastic compound mixture on epigenetic transgenerational inheritance of adult onset disease. The *in vivo* doses used in previous studies that did not promote overt toxicity (litter size, sex ratio or weight changes) were selected [11], [21], [22], [23], [24], [25], [26], [27], [28], [29], [30]. The doses used were based on a <1% fraction of the oral LD50 dose for bisphenol-A (1%, 50 mg/kg/day), DEHP (0.025%, 750 mg/kg/day) and DBP (0.8%, 66 mg/kg/day) [11], [21], [22], [23], [24], [25], [26], [27], [28], [29], [30]. A lower dose of half this was also used, which should not be considered “low dose” as previously described [12], but simply a lower dose. The human exposure is estimated for BPA is 1 µg/kg/day, for DEHP is 52 µg/kg/day, and for DBP is approximately 5 µg/kg/day. Although no overt toxicity was observed in the F1 generation animals, the mode of administration and dose used are higher than anticipated environmental exposures so does not allow risk assessment of these compounds from the current study. However, the potential that these chemicals can have biphasic dose curves with lower doses having greater effects needs to be considered and impacts this dose discussion. The objective of the study was to investigate if exposure to the plastics mixture could potentially promote epigenetic transgenerational inheritance of a disease phenotype and not to assess risk of the exposure to these compounds. Future studies with more appropriate mode of administration and dose curves will be required for risk assessment. Observations from the current study will now allow more effective risk assessment studies to be designed.

Transgenerational disease phenotypes are unique in the sense that they are not caused by direct exposure to the environmental chemical. As discussed above, effects of these compounds are primarily assessed in direct exposure studies. When the exposure occurs to a gestating female during the critical period of gonad sex determination, not only the F0 generation female, but also the developing fetus and the fetal germ cells are directly exposed. Therefore, any pathology observed in F0, F1 (via fetal exposure) and F2 (via fetal germ cell exposure) can be caused by the direct exposure. Therefore, the F3 generation animals derived from the exposed F0 generation female are the first generation to clearly demonstrate epigenetic transgenerational inheritance of disease phenotypes [31]. In the current study we examined the pathology in F1 generation animals to observe any direct epigenetic effects on somatic tissues and in the F3 generation animals to observe germline mediated epigenetic transgenerational effects. Although similarities in phenotype can occur, the distinct mechanisms involved suggest differences in phenotype are anticipated.

The primary transgenerational disease/abnormality phenotypes observed include testis disease, ovary disease, obesity and pubertal abnormalities. The testicular disease incidence was significantly higher in the F3 generation in the lower dose plastics lineage males at one year of age. The spermatogenic cell apoptosis was also significantly increased in these males which further supports the development of testis disease. In recent years there is a trend of a gradual decline in sperm concentration in most human populations [82] and human male infertility is approaching 10% [83]. The etiology of testicular disease and rise in infertility are suspected to be at least in part due to exposure to environmental chemicals, including endocrine disruptor toxicants [84]. The potential role of epigenetic transgenerational inheritance of male infertility needs to be considered [1]. The testicular disease observed in the F3 generation lower dose plastics lineage males provides support for a role of environmental epigenetics and ancestral exposures in male infertility.

Ovarian disease in the form of primordial follicle loss and polycystic ovarian disease was significantly increased in F3 generation plastics and lower dose plastics lineage females at one year of age. Currently the world's population of women are facing increased ovarian diseases of primary ovarian insufficiency characterized by primordial follicle loss, and polycystic ovarian disease characterized by the presence of anovulatory cystic structures [85], [86]. Polycystic ovarian disease is now one of the most common reproductive diseases in the human female population [87]. Similar to the testicular disease/abnormality, the ovary disease phenotypes in the current study may also be the outcome of epigenetic transgenerational inheritance following ancestral environmental exposures. It is important to note that the ovarian disease observed had an increased frequency both in the directly exposed offspring (F1) and transgenerationally (F3). All females examined in the F3 generation plastics and lower dose plastics lineages had polycystic ovarian disease. In a previous study, observations demonstrated a significant transgenerational alteration in both the transcriptome and the epigenome of the ovarian granulosa cells from rats of the F3 generation vinclozolin lineage [88]. Epigenetic mechanisms have been suggested to underlie the development of polycystic ovary syndrome phenotypes in women [89] and prenatally androgenized rhesus monkeys [90]. In addition to considering the effects of direct exposure, the current study suggests epigenetic mechanisms allow the transmission of the disease to future generation offspring following ancestral exposure to abnormal environmental toxicants. Therefore, ancestral exposure to plastics may contribute to the development of these ovarian diseases. Observations suggest an additional paradigm be considered for the etiology of primary ovarian insufficiency and polycystic ovarian disease in women.

Pubertal abnormalities were significantly increased in the females of the F3 generation plastics and lower dose plastics lineages. Puberty is a milestone in developmental physiology and the axis of hypothalamus-pituitary-gonad shows progressive changes during fetal development and matures in adolescence [91]. In rats there are clear external genital changes that indicate pubertal onset (vaginal opening and balano-preputial separation) [92]. Puberty checks were performed from postnatal day 30 in females and day 35 in males in this study. In an earlier report [20] it was shown that the F3 generation plastics and lower dose plastics lineage females had a significant alteration of the pubertal onset (number of days to pubertal onset) compared to control females. The current study assessed the pubertal abnormalities incidence using a puberty cutoff of mean of controls ± 2 standard deviations. Observations demonstrate F1 generation females and males of plastics and lower dose plastics lineages had increased incidence of delayed pubertal onset. The F3 generation females of plastics and lower dose plastics lineages had increased proportion of early onset of puberty, while males of plastics and lower dose plastics lineages had an increased incidence of delayed onset of puberty. The F1 generation phenotypes are due to direct somatic tissue actions, while the F3 generation is due to germ line mediated transgeneration mechanisms. Pubertal abnormalities, have increased over the past several decades in human populations [91]. The early versus delayed onset of puberty has influences on different adult onset clinical conditions. Early onset of puberty leads to accelerated bone mineralization and short adult height in girls and predisposes them to breast cancers. Delayed onset of puberty leads to decreased bone mineralization, psychological stress and metabolic disease [93]. Previous studies have suggested early onset of puberty in girls is suspected to be caused by environmental exposure to an endocrine disruptor [94]. Early onset of puberty in girls disrupts health by affecting brain development, endocrine organ systems and growth, leading to later increase in susceptibility to disease. Observations of the current study suggest abnormal pubertal onset (an early developmental milestone) is associated with epigenetic transgenerational adult onset ovary disease, primordial follicle loss and polycystic ovaries in the F3 generation females of both plastics and lower dose plastics lineages.

Obesity was significantly increased in the F3 generation females and there was a tendency to be increased in the F3 generation males of the lower dose plastics lineage. Interestingly, the obesity was not observed in the direct exposure F1 generation, but only in the F3 generation suggesting a transgenerational mechanism is involved. Bisphenol-A and phthalates are suspected obesogens [95] and direct exposures have been shown to promote obesity [96]. Obesity is associated with other diseases and clinical conditions including cardiovascular disease, type 2 diabetes, and a diminished average life expectancy [97]. Obesity is a component of a complex disease condition termed metabolic disease syndrome [98]. Obese women have a higher prevalence of amenorrhea and infertility. A major associated disease with obesity is polycystic ovarian disease. The majority of the females with obesity have polycystic ovarian disease [99], [100]. Interestingly, the current study demonstrated the F3 generation plastic lineage females developed both obesity and polycystic ovarian disease. Therefore, the ability of a BPA and phthalates mixture to promote the transgenerational inheritance of obesity and polycystic ovarian disease supports an association of these diseases. Maternal obesity can have a negative effect on children's health [101]. Experimental studies in rats indicate that obese dams are responsible for the appearance of obesity in the subsequent generation [102]. Waterland et al., (2008) [103] suggested that epigenetic mechanisms are involved in this generational transmission of maternal obesity. The current study extends this concept that epigenetic transgenerational inheritance in the absence of any direct exposure may promote obesity. Therefore, ancestral exposure to environmental plastic compounds such as BPA and phthalates may influence adult-onset obesity. Future studies will need to evaluate the adult status of obesity associated conditions such as adiposity, bone mineralization, adult height and metabolic disease in the F3 generation plastics and lower dose plastics lineages. Observations suggest the different disease phenotypes observed (testis disease, ovary disease, pubertal abnormality and obesity) may be linked in a complex disease syndrome that involves an epigenetic transgenerational inheritance etiology.

The molecular mechanism involved in epigenetic transgenerational inheritance of adult-onset disease phenotypes involves reprogramming of the germline (sperm) epigenome during sex determination [1], [5]. The modified sperm epigenome (DNA methylation) appears to become permanently reprogrammed in an imprinted-like manner and is protected from DNA de-methylation and reprogramming after fertilization. This allows transgenerational transmission of the modified sperm epigenome and subsequent modification of somatic cell and tissue epigenomes and transcriptomes [104]. All tissues and cells will have a transgenerational transcriptome [105] and those tissues sensitive to this modified transcriptome will develop disease. Therefore, the current study further examined the altered sperm epigenome and epimutations induced by the plastic compound mixture previously identified [20].

A transgenerational alteration in sperm DNA methylation has been shown to be induced by vinclozolin [2], [4]. A transgenerational change in the fetal testis transcriptome has also shown to be induced by vinclozolin [106]. More recently, all tissues examined in the F3 generation vinclozolin lineage had a tissue specific transgenerational transcriptome [105]. A previous study used F3 generation rat sperm from plastics and control lineages were used for genome wide promoter DNA methylation analysis using an MeDIP-Chip protocol [20]. Differential DNA methylated regions (DMR) defined as epimutations and epigenetic biomarkers were identified for the plastics lineage F3 generation sperm in comparison with control lineage F3 generation sperm [20]. The current study more thoroughly examined these DMR that are presented in Table S4. A DMR was selected and used in a MeDIP-QPCR analysis to confirm the MeDIP-Chip analysis previously reported [20]. The *Gdnf* gene associated DMR selected had a change that confirmed the MeDIP-Chip analysis when the sperm from 120 day old males was investigated. Future analysis will require analysis of age affects and more genome-wide analysis. The DMR chromosomal locations were identified and the gene functional categories for the 197 genes associated with the DMR. A gene network analysis identified a direct connection network between the genes associated with the DMR (Figure 8). These interconnected genes have previously been shown to have direct functional and/or binding associations. Several cellular signaling pathways and processes were identified within the gene network that will be of interest for future investigations. Therefore, the epigenetic analysis confirmed the development of epimutations in the sperm and a role in epigenetic transgenerational inheritance of the disease phenotypes observed.

The altered sperm epigenome will generate altered epigenomes in all the cells generated from the sperm which will be distinct between cell types [104], [105]. The cascade of epigenetic and genetic (transcriptome) changes involved in generating an adult cell type will likely have negligible correlations with the specific original sperm epigenome and associated genes. However, the sperm DMR associated gene regulation may influence developmental events promoting the adult onset disease. The correlation of the DMR associated genes with genes previously shown to be linked to one of the major transgenerational disease phenotypes observed was accomplished. The DMR list had 5 genes previously shown to be associated with the onset of obesity (Figure 9). These included *Tnfrsf12a* [107], *Esrra* [108], *Fgf19* [109], *Wnt10b* [110], and *Gdnf* [111]. Therefore, a number of the epimutation associated genes identified in the F3 generation plastic lineage sperm were found to be linked to the adult onset of obesity. Two DMR associated genes found in both the gene network analysis and obesity associated gene list were *Gdnf* and *Esrra*. Future studies on the various cell types associated with the disease/abnormality phenotypes will be required to determine potential correlations with the sperm DMR identified.

Epigenetic transgenerational inheritance of disease has been shown to be promoted by several environmental compounds [20], [112]. Vinclozolin exposure resulted in F3 generation testis disease, prostate disease, kidney disease, immune system abnormalities, tumors, uterine hemorrhage during pregnancy and polycystic ovary disease [2], [3], [20], [32], [113]. Alterations in methylation patterns of sperm of F3 generation rats and mice have been reported following exposure of F0 generation females to vinclozolin [2], [3], [4], [114]. Exposure of F0 generation gestating rats to bisphenol-A caused decreased fertility in F3 generation males [19]. Transgenerational decline in fertility in F3 generation mice was also documented following exposure to dioxin of gestating F0 generation females [20], [112], [115]. Other environmental factors such as nutrition [103] also can promote epigenetic transgenerational inheritance of disease phenotypes. Demonstration of epigenetic transgenerational inheritance in worms [116], flies [117], plants [118] and mammals [119], [120], [121] suggest this phenomena will likely be critical in biology and disease etiology [1]. Combined observations demonstrate exposure of gestating females during the critical development period of gonadal sex determination to a plastics endocrine disruptor mixture consisting of bisphenol-A, DEHP and DBP promotes epigenetic transgenerational inheritance of adult-onset disease including testis disease, ovarian disease, pubertal abnormalities and obesity. All these disease phenotypes have an impact on fertility and reproduction. The overall increase in total disease and multiple diseases in F3 generation plastics and lower dose plastics lineages is considerable. Associated with the occurrence of these transgenerational diseases are the epigenetic changes in rat sperm DNA. These epimutations may be useful as early stage biomarkers of compound exposure and adult onset disease. Although not designed for risk assessment, these findings have implications for the human population that is exposed to these compounds and is experiencing significant decline in fertility and incidence of adult onset disease.

## Materials and Methods

### Animal Studies

All experimental protocols for the procedures with rats were approved by the Washington State University Animal Care and Use Committee (IACUC) (approval # 02568-026). Washington State University Department of Environmental Health and Safety approved the protocols for the use of environmental chemicals. Female and male rats of an outbred strain Sprague Dawley SD (Harlan) of about 70 and 100 days of age were maintained in ventilated isolator cages containing Aspen Sani-chips. Rats were fed ad libitum with a standard rat diet and ad libitum tap water for drinking. During the injection, vaginal smear collection, weaning and puberty checking procedures rats were held in an animal transfer station. To obtain time-pregnant females the female rats in proestrus were pair-mated with male rats. The sperm-positive (day 0) rats were considered pregnant and monitored for diestrus and body weight. On embryonic day 8 (E8) through E14 of gestation [113], the gestating females were administered daily intraperitoneal injections of the plastic compound mixture (BPA 50 mg/kg BW/day, DEHP 750 mg/kg BW/day and DBP 66 mg/kg/BW/day) or dimethyl sulfoxide (DMSO) (vehicle) with an equal volume of sesame oil (Sigma) to prevent irritation at the injection site. The gestating females rats treated with vehicle or plastic compound mixture were designated as the F0 generation. When selected litters from the plastics lineage F1 generation litter size and sex ratio were reduced, another treatment lineage with exactly half of the original dose for each compound was generated and it was designated “lower dose plastics.” These treatment lineages are designated “control”, “plastics” (bisphenol-A, DEHP and DBP mixture) or “lower dose plastics” lineages throughout the manuscript. The number of animals used for each generation and exposure lineage are outlined in Tables S2 and S3. For female F1 generation total were control (20 animals), plastics (17 animals), lower dose plastics (35 animals), and for male F1 generation the total were control (22 animals), plastics (14 animals), lower dose plastics (46 animals), and for female F3 generation totals were control (69 animals), plastics (43 animals), lower dose plastics (52 animals), and for male F3 generation totals were control (56 animals), plastics (40 animals), and lower dose plastics (58 animals). The animals per litter (litter representation) mean ± SEM for each specific disease/abnormality assessment between the control and plastic or lower dose plastic lineages was not found to be statistically different (p>0.05), so no litter bias was identified.

### Breeding

The offspring of the F0 generation rats were the F1 generation. Non-littermate females and males aged 70–90 days from F1 generation control or plastics or low dose plastics lineages were bred to obtain F2 generation offspring. The F2 generation rats were bred to obtain F3 generation offspring. No sibling or cousin breeding was used to avoid any inbreeding artifacts. Suckling rats were weaned from their mothers at 21 days of age. It is important to note that only the F0 generation gestating female was exposed directly to the control vehicle or plastics or low dose plastics treatment, and the F1–F3 generations were not subjected to any treatment.

### Tissue Harvest and Histology Processing

One-year old rats were euthanized by CO_2_ inhalation for tissue harvest. Body and organ weights were measured at dissection time. Testis, epididymis, prostate, seminal vesicle, ovaries, uterus and kidney were collected and fixed in Bouin's solution (Sigma) and 70% ethanol, then processed for paraffin embedding by standard procedures for histopathology examination. Five-micrometer tissue sections were made and were either unstained and used for TUNEL analysis or stained with H & E stain and examined for histopathology. Blood samples were collected at the time of dissection, allowed to clot, centrifuged and serum samples stored at −20°C for steroid hormone assays.

### Testicular Apoptotic Cell Analysis

Testis sections were examined by a terminal deoxynucleotidyl transferase-mediated dUTP nick end labeling (TUNEL) assay (In situ cell death detection kit, Fluorescein, Roche Diagnostics, Mannheim, Germany) as per the manufacturer's protocols. Sections were deparaffinized and rehydrated. They were deproteinized by Proteinase K (20 mg/ml; Invitrogen, Carlsbad, CA) and then washed with PBS and then 25 µl of the enzyme-label solution mix was applied on the testis sections and incubated at 37°C for 90 min. After PBS washes slides were mounted and kept at 4°C until examination in a fluorescent microscope in dark field. Both testis sections of each slide were microscopically examined to identify and to count apoptotic germ cells by their bright fluorescence.

### Histopathology Examination and Disease/Abnormality Classification

Three different observers examined each unmarked tissue slide and identical criteria were applied to identify diseased tissue. A cut-off was established to declare a tissue ‘diseased’ based on the mean number of histopathological abnormalities plus two standard deviations from the mean number of abnormalities in control tissues by each of the three individual observers. This number was used to classify rats into those with and without disease in testis, prostate or kidney in each lineage. A rat tissue section was declared ‘diseased’ only when at least two of the three observers marked the same tissue section as such. Necropsy and histopathology examinations on rats that died prior to 1 year of age and also pathology analysis of tissues sent with unknown or suspected diseases were performed by the WSU Washington Disease Diagnostic Laboratory and these results were also included in the study. The proportion of rats with obesity or tumor development was obtained by counting those that had these conditions out of all the animals evaluated.

A marked central portion of each prostate, kidney and testis section was microscopically examined under 200x magnification. An additional peripheral portion of each testis section was also examined. Testis histopathology criteria included the presence of a vacuole, azoospermic atretic seminiferous tubule and ‘other’ abnormalities including sloughed spermatogenic cells in center of the tubule and a lack of a tubule lumen. Prostate histopathology criteria included the presence of vacuoles, atrophic epithelial layer of ducts and hyperplasia of prostatic duct epithelium. Kidney histopathology criteria included reduced size of glomerulus, thickened Bowman's capsule and the presence of proteinaceous fluid-filled cysts.

Ovary sections were stained with hematoxylin and eosin and three stained sections (150 µm apart) through the central portion of the ovary with several of the largest cross sections evaluated. Ovary sections were assessed for two diseases, primordial follicle loss and polycystic ovary disease. Primordial follicle loss was determined by microscopically counting the number of primordial follicles per ovary section. Primordial follicle loss was considered present in the ovary when the primordial follicle number was less than the control mean minus two standard deviations. Polycystic ovaries were determined by microscopically counting the number of small cystic structures. The mean number of primordial follicles and small cysts was calculated from three sections. Polycystic ovary disease was considered present when the number of cysts per section was more than the control mean plus two standard deviations. Follicles had to be non-atretic and showing an oocyte nucleus in order to be counted. Primordial follicles had an oocyte surrounded by a single layer of either squamous or both squamous and cuboidal granulosa cells [122], [123]. Cysts were defined as fluid-filled structures of a specified size that were not filled with red blood cells, had no oocyte and negligible granulosa cells. A single layer of cells may line cysts. Small cysts were 50 to 250 µm in diameter measured from the inner cellular boundary across the longest axis. Percentages of females with primordial follicle loss or polycystic ovarian disease were computed.

Onset of puberty was assessed in females by daily examination for vaginal opening from 30 days of age and in males by balano-preputial separation from 35 days of age. For identifying a rat with a pubertal abnormality (either an early or delayed onset of puberty) a mean from all the rats from the control lineage evaluated for pubertal onset was computed and its standard deviation calculated. A range of normal pubertal onset was chosen based on mean ± 2 standard deviations. Any rat with a pubertal onset below this range was considered to have had an early pubertal onset and any rat with a pubertal onset above this range was considered to have had a delayed pubertal onset and the proportion of rats with pubertal abnormality was computed from the total number of rats evaluated for puberty onset.

Obesity was assessed with an analysis of body weight and gross evaluation of abdominal adiposity. The increased fat deposition in an obese animal required presence on most organs (Figure 4D) compared to an absence in non-obese animals. The designation of obesity required the increased body weight and increased abdominal adiposity to be designated obese. Subsequently the correlation to the presence of polycystic ovarian disease was made.

A table of the incidence of individual diseases/abnormalities in rats from each group was created and the proportions of individual disease, Tables S2 and S3, total disease and multiple disease incidences were computed from this table. For the individual disease/abnormality, only those rats that showed a plus (presence of disease) or minus (absence of disease) in the table are included in the computation. Those without a (+) or (−) were not analyzed for that disease. For the total diseases, a column with total disease is presented and the number of plus signs (indicating the presence of disease) were added up for each of the rats and the proportion was computed as the number of rats with one or more diseases (total disease) out of all listed rats. For the multiple diseases, the proportion was computed as the number of rats with more than one disease/abnormality out of all of the listed rats. Not all the rats were evaluated for all diseases/abnormalities due to technical limitations. The computation of the percent incidence of disease data is limiting in this respect and the data presented represent only the minimal incidence of total or multiple disease. For example, if more animals in the current set had been evaluated for ovarian disease, there could have been a higher incidence of either total disease or multiple disease.

### Epididymal Sperm collection

The epididymis was dissected free of connective tissue, the fat pad, the muscles and the vas deferens. A small cut was made to the cauda epididymis and the tissue was placed in 5 ml F12 culture medium containing 0.1% bovine serum albumin for 10 minutes at 37°C and then kept at 4°C to immobilize the sperm. The epididymal tissue in the buffer was put on a petri dish and minced with a blade to release the sperm into the medium, the sperm released into the buffer was aspirated with a pipette into a 1.5 ml centrifuge tube and then centrifuged at 13,000× *g* to pellet the sperm. Sperm were stored in fresh NIM buffer (Nucleus Isolation Medium: 123.0 mmol/l KCl, 2.6 mmol/l NaCl, 7.8 mmol/l NaH_2_PO_4_, 1.4 mmol/l KH_2_PO_4_ and 3 mmol/l EDTA (disodium salt) at −20°C until processed further.

### Sperm methylated DNA immunoprecipitation (MeDIP)

Sperm heads were separated from tails through sonication following a previously described protocol (without protease inhibitors) [124] and then purified using a series of washes and centrifugations [125] from a total of nine F3 generation rats per lineage (control or plastics) that were 120 days of age. DNA extraction on the purified sperm heads was performed as previously described [4]. The same concentrations of DNA from individual sperm samples were then used to produce pools of DNA material. Three DNA pools were produced in total per treatment, each one containing the same amount of sperm DNA from three different animals. Therefore a total of 18 animals were used for building three DNA pools per treatment (control or plastics) making the following groups: C1–C3 and P1–P3. These DNA pools were then used for chromatin immunoprecipitation of methylated DNA fragments (MeDIP). MeDIP was performed as follows: 6 µg of genomic DNA was subjected to series of three 20 pulse sonications at 20% amplitude and the appropriate fragment size (200–1000 ng) was verified through 2% agarose gels; the sonicated genomic DNA was resuspended in 350 µl TE buffer and denatured for 10 min at 95°C and then immediately placed on ice for 5 min; 100 µl of 5X IP buffer (50 mM Na-phosphate pH 7, 700 mM NaCl, 0.25% Triton X-100) was added to the sonicated and denatured DNA. An overnight incubation of the DNA was performed with 5 µg of antibody anti-5-methylCytidine monoclonal from Diagenode (Denville, NJ) at 4°C on a rotating platform. Protein A/G beads from Santa Cruz were prewashed on PBS-BSA 0.1% and resuspended in 40 µl 1X IP buffer. Beads were then added to the DNA-antibody complex and incubated 2 h at 4°C on a rotating platform. Beads bound to DNA-antibody complex were washed 3 times with 1 ml 1X IP buffer; washes included incubation for 5 min at 4°C on a rotating platform and then centrifugation at 6000 rpm for 2 min. Beads-DNA-antibody complex were then resuspended in 250 µl digestion buffer (50 mM Tris HCl pH 8, 10 mM EDTA, 0.5% SDS) and 3.5 µl of proteinase K (20 mg/ml) was added to each sample and then incubated overnight at 55°C on a rotating platform. DNA purification was performed first with phenol and then with chloroform:isoamyl alcohol. Two washes were then performed with 70% ethanol, 1 M NaCl and glycogen. MeDIP selected DNA was then resuspended in 30 µl TE buffer.

### Tiling Array MeDIP-Chip Analysis and MeDIP-QPCR Analysis

The MeDIP-Chip analysis was previously performed and reported [20] and the data used in the current study. Roche Nimblegen's Rat DNA Methylation 3×720 K CpG Island Plus RefSeq Promoter Array was used, which contains three identical sub-arrays, with 720,000 probes per sub-array, scanning a total of 15,287 promoters (3,880 bp upstream and 970 bp downstream from transcription start site). Probe sizes range from 50–75 mer in length with the median probe spacing of 100 bp. Three different comparative (MeDIP vs. MeDIP) hybridizations experiments were performed (3 sub-arrays) for plastics lineage versus control, with each subarray encompassing DNA samples from 6 animals (3 each from plastics and control). MeDIP DNA samples from experimental groups were labeled with Cy3 and MeDIP DNA samples from the control lineage were labeled with Cy5 [20].

The MeDIP samples control and vinclozolin lineage F3 generation sperm were used in an MeDIP-QPCR analysis to confirm the MeDIP-Chip data for a selected gene. A standard RealTime PCR procedure was used to quantify the amount of DNA for the DMR in the MeDIP samples, as previously shown [112]. The PCR primers designed for the genomic DNA sites of the DMR for Gdnf are: 5′ATCCGAGCCTAACTTGCCTG, 3′AGAGTGGAGACCTTTTGCGG. The Q-PCR used 30 cycles and PCR products were quantified and the fold change determined between the F3 generation sperm for control versus plastic lineage MeDIP samples. Statistical analysis of the data used a U-Mann Whitney analysis.

### Bioinformatic and Statistic Analyses of Chip Data

For each comparative hybridization experiment raw data from both the Cy3 and Cy5 channels were imported into R (R Development Core Team (2010), R: A language for statistical computing, R Foundation for Statistical Computing, Vienna, Austria. ISBN 3-900051-07-0, URL http://www.R-project.org), checked for quality and converted to MA values (M =  Cy5−Cy3; A =  (Cy5+Cy3)/2). The normalization procedure as previously described [20]. Following normalization each adjacent >3 probe set value represents the median intensity difference between plastics F3 generation lineage and control F3 generation lineage of a 600 bp window. Significance was assigned to probe differences between plastics F3 generation lineage samples and control F3 generation lineage samples by calculating the median value of the intensity differences as compared to a normal distribution scaled to the experimental mean and standard deviation of the normalized data. A Z-score and P-value were computed for each probe from that distribution. In order to assure the reproducibility of the candidates obtained, only the candidates showing significant changes in all of the single paired comparisons were chosen as a having a significant change in DNA methylation between each experimental group and controls. This is a very stringent approach to select for changes because it only considers repeated changes in all paired analysis. The statistically significant differential DNA methylated regions (DMR) were identified and P-value associated with each region presented, as previously described [20].

Associations between genes (gene networks) containing DMR and particular physiologic cellular processes were determined by an automated, unbiased survey of published literature using Pathway Studio^TM^ software (Ariadne, Elsevier Inc., USA). The specific disease associated genes were also assessed with the Pathway Studio software. Signaling pathway enrichment with genes containing DMR was determined by querying the library of KEGG pathways (Kyto Encyclopedia of Genes and Genomes, http://www.genome.jp/keff/pathway.html).

### Statistical Analysis of Rat Organ and Disease Data

The number of animals or samples for different experiments are presented in the appropriate legends and Tables S2 and S3. For statistical analysis for all data on body and organ weights were used as input in the program GraphPad© Prism 5 statistical analysis program and t-tests were used to determine if the data from the plastics or lower dose plastics group differed from those of control groups. For the number of rats with disease/abnormalities (disease incidence) a logistic regression analysis was used to analyze the data (control or plastics or lower dose plastics, and diseased or unaffected). For the MeDIP-PCR analysis a Student's t-test was utilized. All treatment differences were considered significant if P-value was less than 0.05.

## Supporting Information

Figure S1 Testicular spermatogenic cell apoptosis. Assessed by Terminal deoxynucleotidyl transferase dUTP nick end labeling (TUNEL) in F1 and F3 generation control lineage (open bars), plastics lineage (black bars) and lower dose (LD) plastics lineage (gray bars) rats. Number of apoptotic germ cells were normalized to control means. The mean ± SEM for three different experiments are presented with related difference from control indicated (* P<0.05; *** P<0.001).
(PDF)

Figure S2 Steroid hormone analysis in F3 generation animals. A. Serum estradiol concentrations in proestrus-estrus in F3 generation control, plastics and lower dose (LD) plastics lineage females. B. Serum estradiol concentrations in diestrus in F3 generation females of control, plastics and lower dose (LD) plastics lineages. C. Serum testosterone concentrations in F3 generation males of control, plastics and lower dose (LD) plastics lineages. There were no significant changes (p>0.05) in any of the hormone concentrations of F3 generation rats of plastics and lower dose plastics lineages.
(PDF)

Table S1 **S1A**. Body weight and organ weights in control, plastics and lower dose plastics F1 and F3 generation female rats (Mean ± Standard Error). Asterisks (*, ***), if present, indicate statistically significant differences between means of control and plastics or low dose plastics groups' rats (P<0.05, and P<0.001 respectively). **S1B**. Body weight (grams) and organ weights (% of body weight) in control, plastics and lower dose plastics F1 and F3 generation male rats (Mean ± SE). Asterisks (*, **), if present, indicate statistically significant differences between means of control and plastics or low dose plastics groups' rats (P<0.05, P<0.01 respectively).
(PDF)

Table S2 **S2A**. Individual disease incidence in F1 generation control, plastics and lower dose plastics female rats. The ‘+’ indicates the presence; the ‘−’ indicates the absence of disease; the blank cell “no mark” indicates not determined. Animal IDs with a ‘C’ belong to Control group, those with a ‘P’ belong to plastics group and those with a ‘LP’ belong to lower dose plastics group. PFL  =  Primordial follicle loss; PCO =  Polycystic ovarian disease. See ‘Materials and Methods’ section for disease assessment in rats. **S2B**. Individual disease incidence in F1 generation control, plastics, and lower dose plastics male rats. The ‘+’ indicates the presence; the ‘−’ indicates the absence of disease; the blank cell “no marks” indicates not determined. Animal IDs with a ‘C’ belong to control group, those with a ‘P’ belong to plastics group and those with a ‘LP’ belong to lower dose plastics group. See ‘Materials and Methods’ section for disease assessment in rats. The number of animals per litter (litter representation) mean ± SEM used for each specific disease/abnormality assessment between the control versus plastic or lower dose plastic lineages were not found to be statistically different (p>0.05), so no litter bias detected.
(PDF)

Table S3 **S3A**. Individual disease incidence in F3 generation control, plastics and lower dose plastics female rats. The ‘+’ indicates the presence and the ‘−’ indicates the absence of disease; and blank cell “no mark” indicates not determined. Animal IDs with a ‘C’ belong to Control group, those with a ‘P’ belong to Plastics group, and those with a ‘LP’ belong to lower dose plastics group. See ‘Materials and Methods’ section for disease assessment in rats. **S3B**. Individual disease incidence in F3 generation control, plastics and lower dose plastics male rats. The ‘+’ indicates the presence; the ‘−’ indicates the absence of disease; and blank cell “no mark” indicates not determined. Animal IDs with a ‘C’ belong to Control group, those with a ‘P’ belong to Plastics group, and those with a ‘LP’ belong to Lower Dose Plastics group. See ‘Materials and Methods’ section for disease assessment in rats. The number of animals per litter (litter representation) mean ± SEM used for each specific disease/abnormality assessment between the control versus plastic or lower dose plastic lineages were not found to be statistically different (p>0.05), so no litter bias detected.
(PDF)

Table S4 List of rat sperm differential DNA methylation regions (DMR) found in F3-generation plastic lineage sperm. The functional gene category is presented, chromosomal number, start and stop genome nucleotide location, gene ID, statistical p-value for identified DMR, and name of the gene are presented.
(PDF)

Table S5 The F3 generation plastic lineage sperm DMR associated genes correlation to KEGG pathways. The pathway name, the number of DMR genes, and total number of genes in the pathway are listed.
(PDF)
