# Supplementary figures and images for: Plastics Derived Endocrine Disruptors (BPA, DEHP and DBP) Induce Epigenetic Transgenerational Inheritance of Obesity, Reproductive Disease and Sperm Epimutations
Source: PLoS One. 2013 Jan 24;8(1):e55387. doi: 10.1371/journal.pone.0055387 (PMC3554682; doi:10.1371/journal.pone.0055387)

Supplemental Figure S1

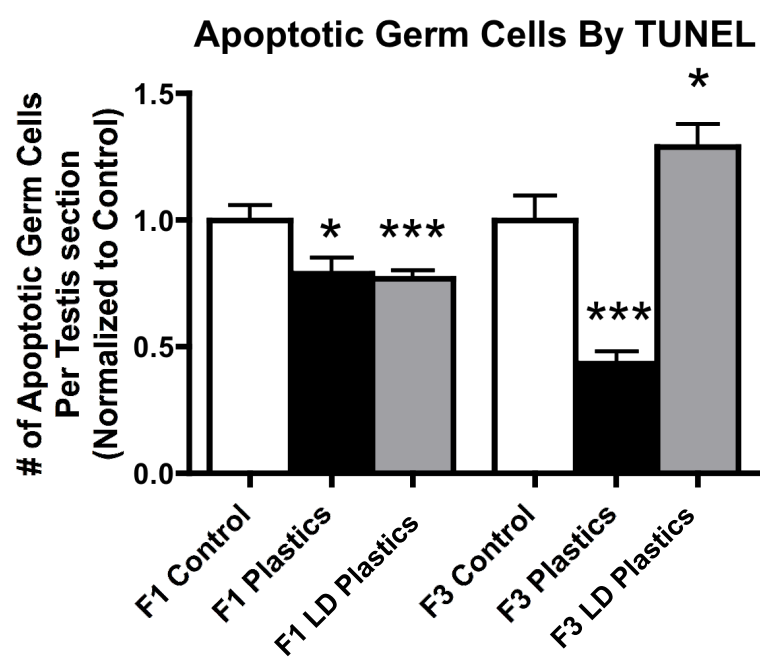

Supplement: Figure S1 — Testicular spermatogenic cell apoptosis. Assessed by Terminal deoxynucleotidyl transferase dUTP nick end labeling (TUNEL) in F1 and F3 generation control lineage (open bars), plastics lineage (black bars) and lower dose (LD) plastics lineage (gray bars) rats. Number of apoptotic germ cells were normalized to control means. The mean ± SEM for three different experiments are presented with related difference from control indicated (* P<0.05; *** P<0.001). (PDF) [file pone.0055387.s001.pdf]

Supplemental Figure S2

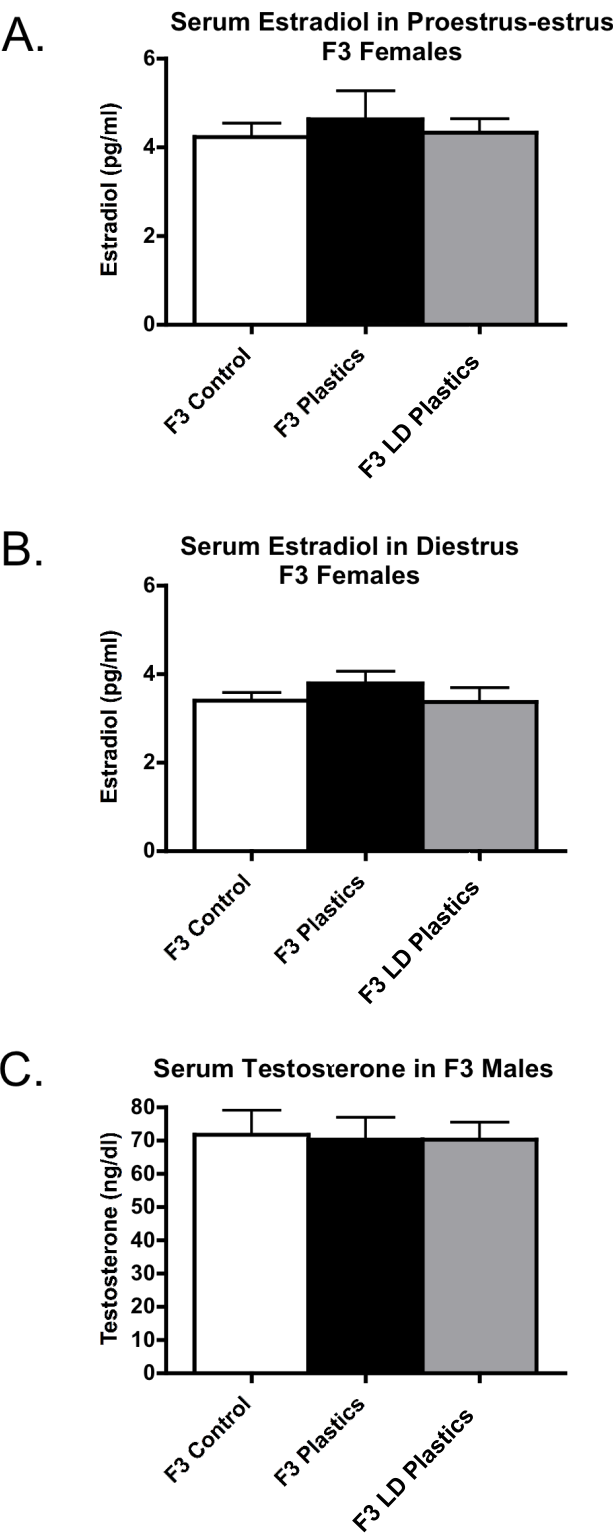

Supplement: Figure S2 — Steroid hormone analysis in F3 generation animals. A. Serum estradiol concentrations in proestrus-estrus in F3 generation control, plastics and lower dose (LD) plastics lineage females. B. Serum estradiol concentrations in diestrus in F3 generation females of control, plastics and lower dose (LD) plastics lineages. C. Serum testosterone concentrations in F3 generation males of control, plastics and lower dose (LD) plastics lineages. There were no significant changes (p>0.05) in any of the hormone concentrations of F3 generation rats of plastics and lower dose plastics lineages. (PDF) [file pone.0055387.s002.pdf]
