# Supplementary material for: Plastics Derived Endocrine Disruptors (BPA, DEHP and DBP) Induce Epigenetic Transgenerational Inheritance of Obesity, Reproductive Disease and Sperm Epimutations
Source: PLoS One. 2013 Jan 24;8(1):e55387. doi: 10.1371/journal.pone.0055387 (PMC3554682; doi:10.1371/journal.pone.0055387)
Supplement: Table S1 — S1A. Body weight and organ weights in control, plastics and lower dose plastics F1 and F3 generation female rats (Mean ± Standard Error). Asterisks (*, ***), if present, indicate statistically significant differences between means of control and plastics or low dose plastics groups' rats (P<0.05, and P<0.001 respectively). S1B. Body weight (grams) and organ weights (% of body weight) in control, plastics and lower dose plastics F1 and F3 generation male rats (Mean ± SE). Asterisks (*, **), if present, indicate statistically significant differences between means of control and plastics or low dose plastics groups' rats (P<0.05, P<0.01 respectively). (PDF) [file pone.0055387.s003.pdf]

Supplemental Table S1A. Body Weight and Organ Weights in Control, Plastics and Lower Dose Plastics F1 and F3 Generation Female Rats (Mean ± Standard Error).

Generation	Treatment	Sex	Body Weight (grams)	Ovaries (% BW)	Uterus (% BW)	Kidney (% BW)
F1	Control	F	310.9 ± 6.15	0.0532 ± 0.0032	0.2589 ± 0.0209	0.3276 ± 0.0127
F1	Plastics	F	300.0 ± 5.83	0.0489 ± 0.0036	0.3143 ± 0.0289	0.3469 ± 0.0077
F1	Lower Dose Plastics	F	290.7 ± 3.91	0.0374* ± 0.0035	0.4440*** ± 0.0302	0.3551 ± 0.0079
F3	Control	F	293.6 ± 2.31	0.0501 ± 0.0011	0.3883 ± 0.0150	0.3567 ± 0.0036
F3	Plastics	F	293.6 ± 3.25	0.04796 ± 0.0017	0.3421* ± 0.0135	0.3504 ± 0.0040
F3	Lower Dose Plastics	F	293.4 ± 3.02	0.0502 ± 0.0016	0.3201* ± 0.0118	0.3589 ± 0.0049

Supplemental Table S1B. Body Weight (grams) and Organ Weights (% of body weight) in Control, Plastics and Lower Dose Plastics F1 and F3 generation Male Rats (Mean ± SE).

Generation	Treatment	Sex	Body Weight (grams)	Testis (% BW)	Prostate (% BW)	Seminal Vesicle (% BW)	Epididymis (% BW)	Kidney (% BW)
F1	Control	M	545.3	0.7599	0.2239	0.1395	0.2585	0.3821
			± 8.66	± 0.0154	± 0.0076	± 0.0092	± 0.0045	± 0.0092
F1	Plastics	M	552.4	0.7788	0.2221	0.1237	0.2574	0.3629
			± 9.54	± 0.0235	± 0.0063	± 0.0078	± 0.0051	± 0.0154
F1	Lower Dose Plastics	M	557.2	0.7683	0.2002	0.1197	0.2494	0.3738
			± 9.44	± 0.0168	± 0.0079	± 0.0057	± 0.0059	± 0.0130
F3	Control	M	515.80	0.8153	0.1970	0.1330	0.2628	0.3791
			± 6.09	± 0.0102	± 0.0041	± 0.0038	± 0.0028	± 0.0010
F3	Plastics	M	538.0*	0.8040	0.1886	0.1203*	0.2604	0.3467
			± 5.33	± 0.0094	± 0.0044	± 0.0039	± 0.0036	± 0.0055
F3	Lower Dose Plastics	M	512.8	0.8080	0.1883	0.1152**	0.2721*	0.3818
			± 4.66	± 0.0130	± 0.0041	± 0.0048	± 0.0034	± 0.0055
