# Supplementary material for: Plastics Derived Endocrine Disruptors (BPA, DEHP and DBP) Induce Epigenetic Transgenerational Inheritance of Obesity, Reproductive Disease and Sperm Epimutations
Source: PLoS One. 2013 Jan 24;8(1):e55387. doi: 10.1371/journal.pone.0055387 (PMC3554682; doi:10.1371/journal.pone.0055387)
Supplement: Table S2 — S2A. Individual disease incidence in F1 generation control, plastics and lower dose plastics female rats. The ‘+’ indicates the presence; the ‘−’ indicates the absence of disease; the blank cell “no mark” indicates not determined. Animal IDs with a ‘C’ belong to Control group, those with a ‘P’ belong to plastics group and those with a ‘LP’ belong to lower dose plastics group. PFL = Primordial follicle loss; PCO = Polycystic ovarian disease. See ‘Materials and Methods’ section for disease assessment in rats. S2B. Individual disease incidence in F1 generation control, plastics, and lower dose plastics male rats. The ‘+’ indicates the presence; the ‘−’ indicates the absence of disease; the blank cell “no marks” indicates not determined. Animal IDs with a ‘C’ belong to control group, those with a ‘P’ belong to plastics group and those with a ‘LP’ belong to lower dose plastics group. See ‘Materials and Methods’ section for disease assessment in rats. The number of animals per litter (litter representation) mean ± SEM used for each specific disease/abnormality assessment between the control versus plastic or lower dose plastic lineages were not found to be statistically different (p>0.05), so no litter bias detected. (PDF) [file pone.0055387.s004.pdf]

Supplemental Table S2A. Individual Disease Incidence in F1 Generation Control, Plastics and Lower Dose Plastics Female Rats.

Animal	Animal ID	Sex	Age	Puberty	PFL	PCO	Kidney	Tumor	Obesity	Total Disease
C1	DCS0-1-1-1	F	1 Yr	-	-	-	-	-	-	
C2	DCS0-1-1-2	F	1 Yr	-			-	-	-	
C3	DCS0-1-1-3	F	1 Yr	+			+	-	-	2
C4	DCR1-1-2-1	F	1 Yr	+	-	-	-	-	-	1
C5	DCR1-1-2-2	F	1 Yr	+			-	-	-	1
C6	DCR1-1-2-3	F	1 Yr	+				+	-	2
C7	DCB1-1-3-1	F	1 Yr	-			-	-	-	
C8	DCB1-1-3-2	F	1 Yr	-				-	-	
C9	DCF1-1-4-1	F	1 Yr	+	-	-	-	+	-	2
C10	DCF1-1-4-2	F	1 Yr	-	-	-	-	-	-	
C11	DCF1-1-4-3	F	1 Yr	+	-	-	+	-	-	2
C12	DCL1-1-5-1	F	1 Yr	-	+	-	+	-	-	2
C13	DCL1-1-5-2	F	1 Yr	-			-	-	-	
C14	DCL1-1-5-3	F	1 Yr	-	-	-	-	-	-	
C15	DCM2-1-6-1	F	1 Yr	-	-	-	-	-	-	
C16	DCM2-1-6-2	F	1 Yr	+			-	-	-	1
C17	DCM2-1-6-3	F	1 Yr	+	-	-	-	-	-	1
C18	DCF0-1-7-1	F	1 Yr	-			-	-	-	
C19	DCF0-1-7-2	F	1 Yr	-			-	-	-	
C20	DCF0-1-7-3	F	1 Yr	-			-	-	-	
P1	DBL2-1-1-1	F	1 Yr	-	+	-	+	-	-	2
P2	DBL2-1-1-2	F	1 Yr	+	+	-	-	-	-	2
P3	DBM1-1-2-1	F	1 Yr	+	+	-	-	-	-	2
P4	DBM1-1-2-2	F	1 Yr	+	+	+	+	-	-	4

Animal	Animal ID	Sex	Age	Puberty	PFL	PCO	Kidney	Tumor	Obesity	Total Disease
P5	DBH1-1-3-1	F	1 Yr	-	-	+	-	-	-	1
P6	DBH1-1-3-2	F	1 Yr	+	+	+	-	-	-	3
P7	DBH1-1-3-3	F	1 Yr	+			-	-	-	1
P8	DBJ0-1-3-1	F	1 Yr	+	+	-	+	-	-	3
P9	DBJ0-1-3-2	F	1 Yr	+	+	+		+	-	4
P10	DBJ0-1-3-3	F	1 Yr	+	-	+	+	-	-	3
P11	DBJ0-1-3-4	F	1 Yr	+			-	-	-	1
P12	DBS1-1-4-1	F	1 Yr	+			-	-	-	1
P13	DBS1-1-4-2	F	1 Yr	+			+	-	-	2
P14	DBS0-1-5-4	F	1 Yr					-	-	
P15	DBS0-1-5-5	F	1 Yr					-	-	
P16	DBS0-1-5-6	F	1 Yr					-	-	
P17	DBS0-1-5-7	F	1 Yr					-	-	
LP1	DLBB1-1-1-1	F	1 Yr	+	+	+	+	-	-	4
LP2	DLBB1-1-1-2	F	1 Yr	-				-	-	
LP3	DLBS0-1-2-1	F	1 Yr	-	+	+	+	-	-	3
LP4	DLBS0-1-2-2	F	1 Yr	-				-	-	
LP5	DLBS0-1-2-3	F	1 Yr	-				-	-	
LP6	DLBS0-1-2-4	F	1 Yr	-				-	-	
LP7	DLBW1-1-3-1	F	1 Yr	-	+	+	+	-	-	3
LP8	DLBW1-1-3-2	F	1 Yr	-				-	-	
LP9	DLBW1-1-3-3	F	1 Yr	-				-	-	
LP10	DLBJ2-1-4-2	F	1 Yr	+				-	-	1
LP11	DLBJ2-1-4-3	F	1 Yr	+			+	-	-	2
LP12	DLBT2-1-5-4	F	1 Yr	+			+	-	-	2

Animal	Animal ID	Sex	Age	Puberty	PFL	PCO	Kidney	Tumor	Obesity	Total Disease
LP13	DLBT2-1-5-5	F	1 Yr	+				-	-	1
LP14	DLBT2-1-5-6	F	1 Yr	+				-	-	1
LP15	DLBT2-1-5-7	F	1 Yr	+				-	-	1
LP16	DLBF2-1-6-1	F	1 Yr	-	+	+	-	-	-	2
LP17	DLBF2-1-6-2	F	1 Yr	-				-	-	
LP18	DLBF2-1-6-3	F	9 m.	+				+	-	2
LP19	DLBT0-1-7-5	F	1 Yr	-	+	+	-	-	-	2
LP20	DLBT0-1-7-6	F	1 Yr	-				-	-	
LP21	DLBT0-1-7-7	F	1 Yr	-				-	-	
LP22	DLBT0-1-7-8	F	1 Yr	+				-	-	1
LP23	DLBT0-1-7-9	F	1 Yr	+				-	-	1
LP24	DLBH0-1-8-1	F	1 Yr	+	+	+	-	-	-	3
LP25	DLBH0-1-8-2	F	1 Yr	-	+	+		-	-	2
LP26	DLBK0-1-9-2	F	1 Yr	-	+	+	-	-	-	2
LP27	DLBK0-1-9-3	F	1 Yr	-	+	+		-	-	2
LP28	DLBJ0-1-10-1	F	1 Yr	-			-	-	-	
LP29	DLBJ0-1-10-2	F	1 Yr	-				-	-	
LP30	DLBJ0-1-10-3	F	1 Yr	-				-	-	
LP31	DLBJ0-1-10-4	F	1 Yr	-				-	-	
LP32	DLBJ0-1-10-5	F	1 Yr	-				-	-	
LP33	DLBA2-1-11-1	F	1 Yr	+			-	-	-	1
LP34	DLBA2-1-11-2	F	1 Yr	+				-	-	1
LP35	DLBB1-1-12-1	F	1 Yr	+			-	-	-	1

A ‘+’ indicates the presence; A ‘-‘ indicates the absence of disease; A blank cell indicates ‘not determined.’ Animal IDs with a ‘C’ belong to Control group, those with a ‘P’ belong to plastics group and those with a ‘LP’ belong to lower dose plastics group. PFL = Primordial follicle loss; PCO= Polycystic ovarian disease. See ‘Materials and Methods’ section for disease assessment in rats. The number of animals per litter (litter representation) mean ± SEM used for each specific disease/abnormality assessment between the control versus plastic or lower dose plastic lineages were not found to be statistically different ($p>0.05$), so no litter bias detected.

Supplemental Table S2B. Individual Disease Incidence in F1 Generation Control, Plastics, and Lower Dose Plastics Male Rats.

Animal	Animal ID	Sex	Age	Puberty	Testis	Prostate	Kidney	Tumor	Obesity	Total Disease
C1	DCS0-1-1-5	M	1 Yr	-	+	-	-	-	-	1
C2	DCS0-1-1-6	M	1 Yr	-	-	-	-	-	-	
C3	DCR1-1-2-5	M	1 Yr	-	-	-	-	-	-	
C4	DCR1-1-2-6	M	1 Yr	-		-	-	-	-	
C5	DCR1-1-2-7	M	1 Yr	-		+	-	-	-	1
C6	DCR1-1-2-8	M	1 Yr	-	-	-	-	-	-	
C7	DCB1-1-3-3	M	1 Yr	-	+	-	-	-	-	1
C8	DCB1-1-3-4	M	1 Yr	-	-	-	-	-	-	
C9	DCF1-1-4-6	M	1 Yr	-	-	-	-	-	-	
C10	DCF1-1-4-7	M	1 Yr	+	-	+	-	-	-	2
C11	DCF1-1-4-8	M	1 Yr	-	-	-	-	-	-	
C12	DCF1-1-4-9	M	1 Yr	+	-	-	-	-	-	1
C13	DCL1-1-5-6	M	1 Yr	-	-	-	-	-	-	
C14	DCL1-1-5-7	M	1 Yr	-	-	+	-	-	-	1
C15	DCL1-1-5-8	M	1 Yr	-	-	-	-	-	-	
C16	DCL1-1-5-9	M	1 Yr	-	-	+	-	-	-	1
C17	DCM2-1-6-7	M	1 Yr	+	-	+	-	-	-	2
C18	DCM2-1-6-9	M	1 Yr	+	+	-	-	-	-	2
C19	DCM2-1-6-10	M	1 Yr	-	+	-	-	-	-	1
C20	DCF0-1-7-5	M	1 Yr	-		-	-	-	-	
C21	DCF0-1-7-6	M	1 Yr	-	-	-	+	-	-	1
C22	DCF0-1-7-7	M	1 Yr	-	-	-	-	-	-	

Animal	Animal ID	Sex	Age	Puberty	Testis	Prostate	Kidney	Tumor	Obesity	Total Disease
P1	DBL2-1-1-6	M	1 Yr	+	-	+	-	-	-	2
P2	DBM1-1-2-4	M	1 Yr	+	+	-	-	-	-	2
P3	DBH1-1-3-5	M	1 Yr	-	-	-	+	-	-	1
P4	DBH1-1-3-6	M	1 Yr	+	+	-	+	-	-	3
P5	DBH1-1-3-7	M	1 Yr	-	-	+	-	-	-	1
P6	DBH1-1-3-8	M	1 Yr	-	-	-	+	-	-	1
P7	DBJ0-1-3-5	M	1 Yr	+	-	-	+	-	-	2
P8	DBJ0-1-3-6	M	1 Yr	+	+	+	+	-	-	4
P9	DBJ0-1-3-7	M	1 Yr	+	+	+		-	-	3
P10	DBS1-1-4-7	M	1 Yr	-	-	+	+	-	-	2
P11	DBS1-1-4-8	M	1 Yr	-	+		-	-	-	1
P12	DBS0-1-5-1	M	1 Yr		+	+	-	-	-	2
P13	DBS0-1-5-2	M	1 Yr		-	+	-	-	-	1
P14	DBS0-1-5-10	M	1 Yr					-	-	
LP1	DLBB1-1-1-3	M	1 Yr	-	+	-	+	-	-	2
LP2	DLBB1-1-1-4	M	1 Yr	+	-			-	-	1
LP3	DLBB1-1-1-5	M	1 Yr	+				-	-	1
LP4	DLBB1-1-1-6	M	1 Yr	+				-	-	1
LP5	DLBB1-1-1-7	M	1 Yr	+				-	-	1
LP6	DLBB1-1-1-8	M	1 Yr	-				-	-	
LP7	DLBB1-1-1-9	M	1 Yr	-	+	+	+	-	-	3
LP8	DLBS0-1-2-5	M	1 Yr	-	+	+	+	-	-	3
LP9	DLBS0-1-2-6	M	1 Yr	-	+		-	-	-	1
LP10	DLBS0-1-2-7	M	1 Yr	-	-			-	-	

Animal	Animal ID	Sex	Age	Puberty	Testis	Prostate	Kidney	Tumor	Obesity	Total Disease
LP11	DLBW1-1-3-4	M	1 Yr	-				-	-	
LP12	DLBW1-1-3-5	M	1 Yr	-	-	-	-	-	-	
LP13	DLBW1-1-3-6	M	1 Yr	-				-	-	
LP14	DLBW1-1-3-7	M	1 Yr	-				-	-	
LP15	DLBW1-1-3-8	M	1 Yr	-				-	-	
LP16	DLBW1-1-3-9	M	1 Yr	-				-	-	
LP17	DLBW1-1-3-10	M	1 Yr	-	-	+	+	-	-	2
LP18	DLBG0-1-4-1	M	1 Yr	-	-	-	+	+	-	2
LP19	DLBJ2-1-4-8	M	1 Yr	-				-	-	
LP20	DLBJ2-1-4-9	M	1 Yr	-				-	-	
LP21	DLBJ2-1-4-11	M	1 Yr	-				-	-	
LP22	DLBT2-1-5-10	M	1 Yr	+				-	-	1
LP23	DLBF2-1-6-4	M	1 Yr	+			+	-	-	2
LP24	DLBF2-1-6-5	M	1 Yr	+	-	+	-	-	-	2
LP25	DLBF2-1-6-6	M	1 Yr	+				-	-	1
LP26	DLBF2-1-6-7	M	1 Yr	+				-	-	1
LP27	DLBF2-1-6-8	M	1 Yr	-				+	-	1
LP28	DLBT0-1-7-12	M	1 Yr	-			-	-	-	
LP29	DLBT0-1-7-13	M	1 Yr	-	-	+	-	-	-	1
LP30	DLBH0-1-8-3	M	1 Yr	-			+	-	-	1
LP31	DLBH0-1-8-4	M	1 Yr	-				-	-	
LP32	DLBH0-1-8-5	M	1 Yr	-	-	+	-	-	-	1
LP33	DLBK0-1-9-10	M	1 Yr	+	-	+	+	-	-	3
LP34	DLBK0-1-9-11	M	1 Yr	+	-	+		-	-	2
LP35	DLBK0-1-9-12	M	1 Yr	+				-	-	1
LP36	DLBK0-1-9-13	M	1 Yr	+				-	-	1

Animal	Animal ID	Sex	Age	Puberty	Testis	Prostate	Kidney	Tumor	Obesity	Total Disease
LP37	DLBK0-1-9-14	M	1 Yr	+	-	-	-	-	-	1
LP38	DLBJ0-1-10-6	M	1 Yr	-	-	-	-	-	-	
LP39	DLBJ0-1-10-7	M	1 Yr	+	-	+	-	-	-	2
LP40	DLBJ0-1-10-8	M	1 Yr	+	-	-	-	-	-	1
LP41	DLBA2-1-11-3	M	1 Yr	+	-	+		-	-	2
LP42	DLBA2-1-11-4	M	1 Yr	+				-	-	1
LP43	DLBA2-1-11-5	M	1 Yr	+				-	-	1
LP44	DLBA2-1-11-6	M	1 Yr	+				-	-	1
LP45	DLBB1-1-12-2	M	1 Yr	-		-	+	-	-	1
LP46	DLBB1-1-12-3	M	1 Yr	-		-		-	-	

A '+' indicates the presence; A '-' indicates the absence of disease; A blank cell indicates 'not determined.' Animal IDs with a 'C' belong to control group, those with a 'P' belong to plastics group and those with a 'LP' belong to lower dose plastics group. See 'Materials and Methods' section for disease assessment in rats. The number of animals per litter (litter representation) mean ± SEM used for each specific disease/abnormality assessment between the control versus plastic or lower dose plastic lineages were not found to be statistically different ($p>0.05$), so no litter bias detected.
