# Supplementary material for: Plastics Derived Endocrine Disruptors (BPA, DEHP and DBP) Induce Epigenetic Transgenerational Inheritance of Obesity, Reproductive Disease and Sperm Epimutations
Source: PLoS One. 2013 Jan 24;8(1):e55387. doi: 10.1371/journal.pone.0055387 (PMC3554682; doi:10.1371/journal.pone.0055387)
Supplement: Table S3 — S3A. Individual disease incidence in F3 generation control, plastics and lower dose plastics female rats. The ‘+’ indicates the presence and the ‘−’ indicates the absence of disease; and blank cell “no mark” indicates not determined. Animal IDs with a ‘C’ belong to Control group, those with a ‘P’ belong to Plastics group, and those with a ‘LP’ belong to lower dose plastics group. See ‘Materials and Methods’ section for disease assessment in rats. S3B. Individual disease incidence in F3 generation control, plastics and lower dose plastics male rats. The ‘+’ indicates the presence; the ‘−’ indicates the absence of disease; and blank cell “no mark” indicates not determined. Animal IDs with a ‘C’ belong to Control group, those with a ‘P’ belong to Plastics group, and those with a ‘LP’ belong to Lower Dose Plastics group. See ‘Materials and Methods’ section for disease assessment in rats. The number of animals per litter (litter representation) mean ± SEM used for each specific disease/abnormality assessment between the control versus plastic or lower dose plastic lineages were not found to be statistically different (p>0.05), so no litter bias detected. (PDF) [file pone.0055387.s005.pdf]

Supplemental Table S3A. Individual Disease Incidence in F3 Generation Control, Plastics and Lower Dose Plastics Female Rats.

Animal	Animal ID	Sex	Age	Kidney	Tumor	Obesity	Total Disease
C1	DCF1-3-1-2	F	1 Yr	-	-	-	
C2	DCF1-3-1-3	F	1 Yr	-	-	-	
C3	DCF1-3-1-4	F	1 Yr	-	-	-	
C4	DCF1-3-1-5	F	1 Yr		-	-	
C5	DCS0-3-2-2	F	1 Yr	-	-	-	
C6	DCS0-3-2-3	F	1 Yr	-	-	-	
C7	DCS0-3-2-4	F	1 Yr		-	-	
C8	DCS0-3-2-5	F	1 Yr		-	-	
C9	DCL1-3-3-2	F	1 Yr	+	-	-	1
C10	DCL1-3-3-3	F	1 Yr		+	-	1
C11	DCB1-3-4-2	F	1 Yr	-	-	-	
C12	DCB1-3-4-3	F	1 Yr	-	-	-	
C13	DCB1-3-4-4	F	1 Yr	-	-	-	
C14	DCB1-3-4-5	F	1 Yr		-	-	
C15	DCB1-3-4-6	F	1 Yr		-	-	
C16	DCS0-3-5-2	F	1 Yr	+	-	-	1
C17	DCS0-3-5-3	F	1 Yr	-	-	-	
C18	DCS0-3-5-4	F	1 Yr	-	-	-	
C19	DCS0-3-5-5	F	1 Yr	-	-	-	
C20	DCS0-3-5-6	F	1 Yr		-	-	
C21	DCS0-3-6-2	F	1 Yr	+	-	-	1
C22	DCS0-3-6-3	F	1 Yr	-	-	-	
C23	DCB1-3-7-2	F	1 Yr		-	-	
C24	DCB1-3-7-3	F	1 Yr	-	-	-	
C25	DCB1-3-7-4	F	1 Yr		-	-	
C26	DCB1-3-7-5	F	1 Yr		-	-	
C27	DCB1-3-7-6	F	1 Yr		-	-	
C28	DCR1-3-8-2	F	1 Yr		-	-	
C29	DCR1-3-8-3	F	1 Yr	-	-	-	
C30	DCR1-3-8-4	F	1 Yr	-	-	-	
C31	DCR1-3-8-5	F	1 Yr		-	-	
C32	DCF1-3-9-2	F	1 Yr		-	-	
C33	DCF1-3-9-3	F	1 Yr	-	-	-	
C34	DCF1-3-9-4	F	1 Yr	-	-	-	
C35	DCF1-3-9-5	F	1 Yr		-	-	
C36	DCB1-3-10-2	F	1 Yr		-	-	
C37	DCB1-3-10-3	F	1 Yr	-	-	-	
C38	DCB1-3-10-4	F	1 Yr	-	-	-	
C39	DCL1-3-11-2	F	10 m.		+	-	1
C40	DCL1-3-11-3	F	1 Yr	-	-	-	
C41	DCL1-3-11-4	F	1 Yr	-	-	-	
C42	DCL1-3-11-5	F	1 Yr	-	-	-	
C43	DCL1-3-11-6	F	1 Yr		-	-	
C44	DCL1-3-11-8	F	11 m.		+	-	1
C45	DCB1-3-12-2	F	1 Yr		-	-	

Animal	Animal ID	Sex	Age	Kidney	Tumor	Obesity	Total Disease
C46	DCB1-3-12-3	F	1 Yr	-	-	-	
C47	DCL1-3-13-1	F	1 Yr	-	-	-	
C48	DCL1-3-13-2	F	1 Yr		-	-	
C49	DCL1-3-13-3	F	1 Yr	-	-	-	
C50	DCS0-3-14-2	F	1 Yr		-	-	
C51	DCS0-3-14-3	F	1 Yr	-	-	-	
C52	DCL1-3-15-2	F	1 Yr	-	-	-	
C53	DCL1-3-15-3	F	1 Yr	+	-	-	1
C54	DCL1-3-15-4	F	1 Yr	+	-	-	1
C55	DCL1-3-15-5	F	1 Yr		-	-	
C56	DCS0-3-16-2	F	1 Yr		-	-	
C57	DCS0-3-16-3	F	1 Yr	-	-	-	
C58	DCS0-3-16-4	F	1 Yr	-	-	-	
C59	DCS0-3-16-5	F	1 Yr		-	-	
C60	DCR1-3-17-1	F	1 Yr		-	-	
C61	DCR1-3-17-2	F	1 Yr	-	-	-	
C62	DCR1-3-17-3	F	1 Yr	-	-	-	
C63	DCR1-3-17-4	F	1 Yr		-	-	
C64	DCS0-3-18-2	F	1 Yr		-	-	
C65	DCS0-3-18-3	F	1 Yr	-	-	-	
C66	DCS0-3-18-4	F	1 Yr	-	-	-	
C67	DCF1-3-19-1	F	1 Yr		-	-	
C68	DCF1-3-19-2	F	1 Yr	-	-	-	
C69	DCF1-3-19-3	F	11 m.		+	-	1
P1	DBJ0-3-1-2	F	7 m.		-	-	
P2	DBJ0-3-1-3	F	1 Yr	-	-	-	
P3	DBJ0-3-1-4	F	7 m.		+	-	1
P4	DBJ0-3-1-5	F	1 Yr	-	-	-	
P5	DBJ0-3-1-6	F	1 Yr	-	-	-	
P6	DBM1-3-2-2	F	1 Yr	-	-	-	
P7	DBM1-3-2-3	F	1 Yr	-	-	-	
P8	DBS1-3-3-2	F	1 Yr	-	-	-	
P9	DBS1-3-3-3	F	1 Yr	+	-	-	1
P10	DBS1-3-3-4	F	1 Yr	-	-	-	
P11	DBJ0-3-4-2	F	1 Yr	+	-	-	1
P12	DBJ0-3-4-3	F	1 Yr	-	-	-	
P13	DBJ0-3-4-4	F	1 Yr		-	-	
P14	DBJ0-3-4-5	F	1 Yr		+	-	1
P15	DBJ0-3-4-6	F	1 Yr		-	-	
P16	DBL2-3-5-2	F	10 m.		-	-	
P17	DBL2-3-5-3	F	1 Yr	-	+	-	1
P18	DBL2-3-5-4	F	1 Yr	+	-	-	1
P19	DBL2-3-5-5	F	1 Yr	-	-	-	
P20	DBM1-3-6-2	F	1 Yr	-	-	-	
P21	DBM1-3-6-3	F	1 Yr	-	-	-	
P22	DBL2-3-7-2	F	1 Yr	-	-	-	
P23	DBL2-3-7-3	F	1 Yr	-	+	-	1

Animal	Animal ID	Sex	Age	Kidney	Tumor	Obesity	Total Disease
P24	DBL2-3-7-4	F	1 Yr	-	-	-	
P25	DBM1-3-8-2	F	1 Yr	-	-	-	
P26	DBM1-3-8-3	F	1 Yr	-	-	-	
P27	DBJ0-3-9-2	F	1 Yr	-	-	-	
P28	DBJ0-3-9-3	F	1 Yr	-	-	-	
P29	DBH1-3-10-3	F	1 Yr	+	-	-	1
P30	DBH1-3-10-4	F	1 Yr	-	-	-	
P31	DBH1-3-10-5	F	1 Yr	-	-	-	
P32	DBM1-3-11-3	F	1 Yr	-	-	-	
P33	DBM1-3-11-4	F	1 Yr	-	-	-	
P34	DBM1-3-11-5	F	1 Yr	-	+	-	1
P35	DBM1-3-11-6	F	1 Yr		-	-	
P36	DBH1-3-12-3	F	1 Yr	-	-	-	
P37	DBH1-3-12-4	F	1 Yr	+	-	-	1
P38	DBH1-3-12-5	F	1 Yr	-	-	-	
P39	DBL2-3-13-2	F	1 Yr	-	-	-	
P40	DBL2-3-13-3	F	1 Yr	+	-	-	1
P41	DBH1-3-14-1	F	10 m.		-	-	
P42	DBH1-3-14-2	F	1 Yr	-	-	-	
P43	DBH1-3-14-3	F	1 Yr	-	-	-	
LP1	DLBF2-3-1-6	F	1 Yr	-	-	-	
LP2	DLBF2-3-1-7	F	1 Yr	-	-	-	
LP3	DLBF2-3-1-8	F	1 Yr	+	-	-	1
LP4	DLBB1-3-2-4	F	1 Yr	-	-	-	
LP5	DLBB1-3-2-5	F	1 Yr	-	-	-	
LP6	DLBB1-3-2-6	F	1 Yr	+	-	-	1
LP7	DLBJ2-3-3-3	F	1 Yr	-	-	-	
LP8	DLBJ2-3-4-4	F	9 m.		-	-	
LP9	DLBJ2-3-4-6	F	1 Yr	-	-	-	
LP10	DLBJ2-3-4-7	F	1 Yr	-	-	-	
LP11	DLBF2-3-5-3	F	9 m.		+	-	1
LP12	DLBF2-3-5-4	F	1 Yr	-	-	-	
LP13	DLBF2-3-5-5	F	1 Yr	-	-	-	
LP14	DLBF2-3-5-6	F	1 Yr	-	-	-	
LP15	DLBH0-3-6-6	F	1 Yr	-	-	-	
LP16	DLBH0-3-6-7	F	1 Yr	-	-	-	
LP17	DLBH0-3-7-7	F	1 Yr	-	-	-	
LP18	DLBH0-3-7-8	F	1 Yr	-	-	-	
LP19	DLBJ2-3-8-4	F	1 Yr	-	-	-	
LP20	DLBW1-3-9-4	F	1 Yr	-	-	-	
LP21	DLBW1-3-9-5	F	1 Yr	-	-	-	
LP22	DLBF2-3-10-6	F	1 Yr	-	-	-	
LP23	DLBF2-3-10-7	F	1 Yr	-	-	+	1
LP24	DLBF2-3-10-8	F	1 Yr	-	-	-	
LP25	DLBS0-3-11-3	F	9 m.		-	-	
LP26	DLBS0-3-11-4	F	1 Yr	-	-	-	
LP27	DLBS0-3-11-5	F	1 Yr	-	-	-	

Animal	Animal ID	Sex	Age	Kidney	Tumor	Obesity	Total Disease
LP28	DLBS0-3-12-4	F	9 m.		-	-	
LP29	DLBS0-3-12-5	F	9 m.		-	-	
LP30	DLBS0-3-12-7	F	1 Yr	-	-	-	
LP31	DLBS0-3-12-8	F	1 Yr	-	-	-	
LP32	DLBS0-3-12-9	F	1 Yr	-	-	-	
LP33	DLBW1-3-13-2	F	9 m.		-	+	1
LP34	DLBW1-3-13-3	F	1 Yr	-	-	-	
LP35	DLBB1-3-14-2	F	9 m.		-	+	1
LP36	DLBB1-3-14-5	F	1 Yr	-	-	-	
LP37	DLBB1-3-14-6	F	1 Yr		-	-	
LP38	DLBB1-3-14-7	F	1 Yr	-	-	-	
LP39	DLBB1-3-15-4	F	1 Yr	-	-	-	
LP40	DLBB1-3-15-5	F	1 Yr	-	-	-	
LP41	DLBB1-3-15-6	F	1 Yr	-	-	-	
LP42	DLBB1-3-16-1	F	1 Yr	-	-	-	
LP43	DLBB1-3-16-4	F	1 Yr	-	-	-	
LP44	DLBJ2-3-17-1	F	1 Yr	-	-	-	
LP45	DLBJ2-3-17-4	F	1 Yr	-	-	-	
LP46	DLBJ2-3-17-5	F	1 Yr	-	-	-	
LP47	DLBW1-3-18-1	F	1 Yr	-	-	-	
LP48	DLBW1-3-18-4	F	1 Yr	-	-	-	
LP49	DLBW1-3-18-5	F	1 Yr	-	-	-	
LP50	DLBW1-3-18-6	F	1 Yr	-	-	-	
LP51	DLBW1-3-19-1	F	1 Yr	-	-	+	1
LP52	DLBW1-3-19-4	F	1 Yr	-	-	+	1

The '+' indicates the presence and the '-' indicates the absence of disease. Animal IDs with a 'C' belong to Control group, those with a 'P' belong to Plastics group, and those with a 'LP' belong to Lower Dose Plastics group. See 'Materials and Methods' section for disease assessment in rats. The number of animals per litter (litter representation) mean ± SEM used for each specific disease/abnormality assessment between the control versus plastic or lower dose plastic lineages were not found to be statistically different ($p>0.05$), so no litter bias detected.

Supplemental Table S3B. Individual Disease Incidence in F3 Generation Control, Plastics and Lower Dose Plastics Male Rats.

Animal	Animal ID	Sex	Age	Testis	Prostate	Kidney	Tumor	Obesity	Total Disease
C1	DCF1-3-1-8	M	1 Yr	+		-	-	-	+
C2	DCF1-3-1-9	M	1 Yr		-	-	-	-	
C3	DCF1-3-1-10	M	1 Yr		-	+	-	-	+
C4	DCS0-3-2-11	M	1 Yr	-	-	-	-	-	
C5	DCS0-3-2-12	M	1 Yr	-	-	-	-	-	
C6	DCS0-3-2-13	M	1 Yr	-		-	-	-	
C7	DCL1-3-3-6	M	1 Yr	-	-		-	-	
C8	DCL1-3-3-7	M	1 Yr	-	-		-	-	
C9	DCB1-3-4-9	M	1 Yr	-	+	-	-	-	1
C10	DCB1-3-4-10	M	1 Yr	-	-	-	-	-	
C11	DCB1-3-4-11	M	1 Yr	-	-	-	-	-	
C12	DCB1-3-4-12	M	1 Yr				-	-	
C13	DCB1-3-4-13	M	1 Yr				-	-	
C14	DCS0-3-5-9	M	1 Yr	-		-	-	-	
C15	DCS0-3-5-10	M	1 Yr	-	-	-	-	-	
C16	DCS0-3-5-11	M	1 Yr	-	-	-	-	-	
C17	DCS0-3-6-6	M	1 Yr	-		-	-	-	
C18	DCS0-3-6-7	M	1 Yr	-	-	-	-	-	
C19	DCS0-3-6-8	M	1 Yr	+	-	-	-	-	1
C20	DCB1-3-7-9	M	1 Yr	-		+	-	-	1
C21	DCB1-3-7-10	M	1 Yr	-	-	-	-	-	
C22	DCB1-3-7-11	M	1 Yr	-	-	-	-	-	
C23	DCR1-3-8-7	M	1 Yr	-	-	-	-	-	
C24	DCR1-3-8-8	M	1 Yr	-	-	-	-	-	
C25	DCF1-3-9-11	M	1 Yr	-	-		-	-	
C26	DCF1-3-9-12	M	1 Yr	-			-	-	
C27	DCF1-3-9-13	M	1 Yr	-			-	-	
C28	DCB1-3-10-9	M	9 m.	-	-	-	+	-	1
C29	DCB1-3-10-10	M	1 Yr		-	-	-	-	
C30	DCB1-3-10-11	M	1 Yr	-	-	-	-	-	
C31	DCB1-3-10-12	M	1 Yr	+	-	-	-	-	1
C32	DCB1-3-10-13	M	11 m.		-		+	-	1
C33	DCL1-3-11-9	M	1 Yr	-		-	-	-	
C34	DCL1-3-11-10	M	1 Yr	-	+	-	-	-	1
C35	DCL1-3-11-11	M	1 Yr	-	-	-	-	-	
C36	DCB1-3-12-6	M	1 Yr	-	-	-	-	-	
C37	DCB1-3-12-7	M	1 Yr	+	+	-	-	-	2
C38	DCB1-3-12-8	M	1 Yr	+	-	-	-	-	1
C39	DCL1-3-13-4	M	1 Yr	-	-	-	-	-	
C40	DCS0-3-14-5	M	1 Yr	-	-	-	-	-	
C41	DCS0-3-14-6	M	1 Yr	-	-		-	-	
C42	DCS0-3-14-7	M	1 Yr	-	-	-	-	-	
C43	DCL1-3-15-7	M	1 Yr	-	-	-	-	-	
C44	DCL1-3-15-8	M	1 Yr	-	+	-	-	-	1

Animal	Animal ID	Sex	Age	Testis	Prostate	Kidney	Tumor	Obesity	Total Disease
C45	DCL1-3-15-9	M	1 Yr	-	-	-	-	-	
C46	DCS0-3-16-9	M	1 Yr	-	-	-	-	-	
C47	DCS0-3-16-10	M	1 Yr	-	-	-	-	-	
C48	DCS0-3-16-11	M	1 Yr	-	-	-	-	-	
C49	DCS0-3-16-13	M	1 Yr				-	-	
C50	DCR1-3-17-6	M	1 Yr		-	-	-	-	
C51	DCS0-3-18-10	M	1 Yr		-	+	-	-	1
C52	DCS0-3-18-11	M	1 Yr	+	-	+	-	-	2
C53	DCS0-3-18-12	M	1 Yr	-		-	-	-	
C54	DCF1-3-19-4	M	1 Yr	-		-	-	-	
C55	DCF1-3-19-5	M	1 Yr	-	-		-	-	
C56	DCF1-3-19-6	M	1 Yr	-	-	-	-	-	
P1	DBJ0-3-1-9	M	1 Yr	-	-	-	-	-	
P2	DBJ0-3-1-10	M	1 Yr	-	-	-	-	-	
P3	DBJ0-3-1-11	M	1 Yr	-	-	+	-	-	1
P4	DBJ0-3-1-12	M	1 Yr				-	-	
P5	DBM1-3-2-6	M	1 Yr	-	-	-	-	-	
P6	DBM1-3-2-7	M	8 m.				+	-	1
P7	DBM1-3-2-8	M	1 Yr	-	-	+	-	-	1
P8	DBM1-3-2-9	M	1 Yr	-	+	-	-	-	1
P9	DBS1-3-3-10	M	1 Yr	-	-	-	-	-	
P10	DBJ0-3-4-9	M	1 Yr	-	-	+	-	-	1
P11	DBJ0-3-4-10	M	1 Yr	-	+	+	-	-	2
P12	DBJ0-3-4-11	M	1 Yr	+	+	-	-	-	2
P13	DBJ0-3-4-13	M	1 Yr				-	-	
P14	DBL2-3-5-8	M	1 Yr	-	-	-	-	-	
P15	DBL2-3-5-9	M	1 Yr	-	-	-	-	-	
P16	DBL2-3-5-10	M	1 Yr	-	-	-	-	-	
P17	DBM1-3-6-5	M	1 Yr	-	-	-	-	-	
P18	DBM1-3-6-6	M	1 Yr	-	-	-	-	-	
P19	DBM1-3-6-7	M	1 Yr	-	-	-	-	-	
P20	DBL2-3-7-9	M	1 Yr	-	-	-	-	-	
P21	DBL2-3-7-10	M	10 m				+	-	1
P22	DBL2-3-7-11	M	1 Yr		-	+	-	-	1
P23	DBM1-3-8-4	M	1 Yr				-	+	1
P24	DBM1-3-8-6	M	1 Yr		-	+	-	-	1
P25	DBM1-3-8-7	M	1 Yr	-	+	-	-	-	1
P26	DBM1-3-8-8	M	1 Yr	-	-	-	-	-	
P27	DBJ0-3-9-5	M	1 Yr	-	-	-	-	-	
P28	DBJ0-3-9-6	M	1 Yr	-	-	-	-	-	
P29	DBJ0-3-9-7	M	1 Yr	-	-	-	-	-	
P30	DBH1-3-10-10	M	1 Yr	-	-	-	-	-	
P31	DBH1-3-10-11	M	1 Yr	-	-	+	-	-	1
P32	DBH1-3-10-12	M	1 Yr	-	+	-	-	-	1
P33	DBM1-3-11-10	M	1 Yr	-	-	-	-	-	
P34	DBM1-3-11-11	M	1 Yr	-	-	-	-	-	

Animal	Animal ID	Sex	Age	Testis	Prostate	Kidney	Tumor	Obesity	Total Disease
P35	DBH1-3-12-13	M	1 Yr	-	+		-	-	1
P36	DBH1-3-12-14	M	1 Yr	-	+	-	-	-	1
P37	DBL2-3-13-6	M	1 Yr	-	-	-	-	-	
P38	DBH1-3-14-5	M	1 Yr	-	-		-	-	
P39	DBH1-3-14-6	M	1 Yr	+	-		-	-	1
P40	DBH1-3-14-7	M	1 Yr	-	-		-	-	
LP1	DLBF2-3-1-12	M	1 Yr	-	-	-	-	-	
LP2	DLBB1-3-2-13	M	1 Yr	-	-	-	-	-	
LP3	DLBB1-3-2-14	M	1 Yr	-	-	-	-	-	
LP4	DLBB1-3-2-15	M	1 Yr	-	-	-	-	-	
LP5	DLBJ2-3-3-7	M	1 Yr	+	-	+	+	-	3
LP6	DLBJ2-3-3-8	M	1 Yr	+	-	-	-	-	1
LP7	DLBJ2-3-4-14	M	1 Yr	+	-	+	-	-	2
LP8	DLBJ2-3-4-15	M	1 Yr	-	-	-	-	-	
LP9	DLBJ2-3-4-16	M	1 Yr	-	+	-	-	-	1
LP10	DLBF2-3-5-12	M	1 Yr	-	-	-	-	-	
LP11	DLBF2-3-5-13	M	1 Yr				+	-	1
LP12	DLBF2-3-5-14	M	1 Yr	-	-	+	-	-	1
LP13	DLBH0-3-6-11	M	1 Yr	-	-	-	-	-	
LP14	DLBH0-3-6-12	M	1 Yr	+	-	-	-	-	1
LP15	DLBH0-3-6-13	M	1 Yr	-	-	-	-	+	1
LP16	DLBL2-3-7-12	M	1 Yr	-	-	-	-	-	
LP17	DLBL2-3-7-13	M	1 Yr	-	-	-	-	-	
LP18	DLBL2-3-7-14	M	1 Yr	-	-	-	-	-	
LP19	DLBJ2-3-8-8	M	1 Yr	-	-	-	-	-	
LP20	DLBJ2-3-8-9	M	1 Yr	+	-	-	-	-	1
LP21	DLBJ2-3-8-10	M	1 Yr	-	+	-	-	-	1
LP22	DLBW1-3-9-8	M	10 m.	-		-	+	-	1
LP23	DLBW1-3-9-9	M	1 Yr	-	-	-	-	-	
LP24	DLBW1-3-9-10	M	1 Yr	-	-	-	-	+	1
LP25	DLBW1-3-9-11	M	1 Yr	-	+	-	-	-	1
LP26	DLBF2-3-10-12	M	1 Yr	-			-	-	
LP27	DLBF2-3-10-13	M	1 Yr	+	-	-	-	-	1
LP28	DLBF2-3-10-14	M	1 Yr	-	-	-	-	-	
LP29	DLBS0-3-11-11	M	1 Yr	-	-	-	-	-	
LP30	DLBS0-3-11-12	M	1 Yr	-	-	-	-	-	
LP31	DLBS0-3-11-13	M	1 Yr	-	-	-	-	-	
LP32	DLBS0-3-11-14	M	1 Yr	+	-	-	-	-	1
LP33	DLBS0-3-12-12	M	9 m.				-	-	
LP34	DLBS0-3-12-13	M	1 Yr	+	+	-	-	-	2
LP35	DLBW1-3-13-7	M	1 Yr	+	-	-	-	+	2
LP36	DLBB1-3-14-14	M	1 Yr	+	-	-	-	+	2
LP37	DLBB1-3-14-15	M	1 Yr	-	+	-	-	-	1
LP38	DLBB1-3-14-16	M	1 Yr	+	-	-	+	-	2
LP39	DLBB1-3-15-8	M	9 m.				-	+	1
LP40	DLBB1-3-15-10	M	1 Yr	+	-	-	-	-	1

Animal	Animal ID	Sex	Age	Testis	Prostate	Kidney	Tumor	Obesity	Total Disease
LP41	DLBB1-3-15-11	M	1 Yr	+	-	-	-	-	1
LP42	DLBB1-3-16-5	M	9 m.				-	-	
LP43	DLBB1-3-16-10	M	1 Yr	-	-	+	-	-	1
LP44	DLBB1-3-16-11	M	1 Yr	-	-	-	-	-	
LP45	DLBJ2-3-17-6	M	1 Yr	-	-	-	-	-	
LP46	DLBJ2-3-17-13	M	1 Yr	+	-	-	-	-	1
LP47	DLBJ2-3-17-14	M	1 Yr	-	-	-	-	-	
LP48	DLBJ2-3-17-15	M	1 Yr	-	-	-	-	-	
LP49	DLBJ2-3-17-16	M	1 Yr	+	-	-	-	-	1
LP50	DLBJ2-3-17-17	M	1 Yr	-	-	-	-	-	
LP51	DLBW1-3-18-7	M	1 Yr	+	-	-	-	-	1
LP52	DLBW1-3-18-10	M	1 Yr	-	-	-	-	-	
LP53	DLBW1-3-18-11	M	1 Yr	+	-	-	-	-	1
LP54	DLBW1-3-18-12	M	1 Yr	+	-	+	-	-	2
LP55	DLBW1-3-19-5	M	1 Yr	+	-	+	-	-	2
LP56	DLBW1-3-19-10	M	1 Yr	-	+	-	-	-	1
LP57	DLBW1-3-19-11	M	1 Yr	+	+	-	-	-	2
LP58	DLBW1-3-19-12	M	1 Yr	-	-	+	-	-	1

The ‘+’ indicates the presence and the ‘-‘ indicates the absence of disease. Animal IDs with a ‘C’ belong to Control group, those with a ‘P’ belong to Plastics group, and those with a ‘LP’ belong to Lower Dose Plastics group. See ‘Materials and Methods’ section for disease assessment in rats. The number of animals per litter (litter representation) mean ± SEM used for each specific disease/abnormality assessment between the control versus plastic or lower dose plastic lineages were not found to be statistically different ($p>0.05$), so no litter bias detected.
