# Supplementary material for: Plastics Derived Endocrine Disruptors (BPA, DEHP and DBP) Induce Epigenetic Transgenerational Inheritance of Obesity, Reproductive Disease and Sperm Epimutations
Source: PLoS One. 2013 Jan 24;8(1):e55387. doi: 10.1371/journal.pone.0055387 (PMC3554682; doi:10.1371/journal.pone.0055387)
Supplement: Table S4 — List of rat sperm differential DNA methylation regions (DMR) found in F3-generation plastic lineage sperm. The functional gene category is presented, chromosomal number, start and stop genome nucleotide location, gene ID, statistical p-value for identified DMR, and name of the gene are presented. (PDF) [file pone.0055387.s006.pdf]

Supplemental Table S4. List of differential methylation regions (DMR) found in F3-generation plastics lineage sperm

Gene Symbol	Chr	Start	Stop	Gene ID	min p-value	Gene Title
**Apoptosis**						
Pdcd11	1	252412922	252413522	309458	1.2E-13	programmed cell death 11
Tnfrsf12a	10	12941335	12942463	302965	3.5E-12	tumor necrosis factor receptor superfamily, member 12a
Higd2a	17	16084041	16084831	290999	6.2E-13	HIG1 hypoxia inducible domain family, member 2A
Cell Cycle						
Cep55	1	242381886	242382486	294074	1.6E-15	centrosomal protein 55kDa
Orc4l	3	29851658	29852473	295596	1.8E-10	origin recognition complex, subunit 4-like (yeast)
Amn1	4	186612094	186612807	302032	7.8E-10	antagonist of mitotic exit network 1 homolog (S. cerevisiae)
Dock6	8	20894272	20895072	367039	9.2E-11	dedicator of cytokinesis 6
Cables1	18	3424659	3425645	307585	8.2E-22	Cdk5 and Abl enzyme substrate 1
**Cytoskeleton-ECM**						
Ldb1	1	251260561	251261161	309447	2.1E-22	LIM domain binding 1
Actn3	1	207491938	207492749	171009	1.9E-09	actinin alpha 3
Actl6a	2	118919059	118919939	361925	1.3E-11	actin-like 6A
Tspan33	4	56583349	56583949	500065	1.5E-14	tetraspanin 33
Actg2	4	117748353	117749060	25365	3.8E-11	actin, gamma 2, smooth muscle, enteric
Tpm2	5	60003516	60004592	500450	2.6E-07	tropomyosin 2, beta
LOC100363366	8	30972488	30973193	100363366	3.9E-10	amyloid beta (A4) precursor-like protein 2-like
Tpm1	8	71356826	71357518	24851	1.4E-13	tropomyosin 1, alpha
Ncam2	11	20423008	20423897	288280	3.8E-09	neural cell adhesion molecule 2
Mcoln1	12	2637728	2638418	288371	1.2E-15	mucolipin 1
Fat1	16	50588806	50589703	83720	3.1E-08	FAT tumor suppressor homolog 1 (Drosophila)
Spock3	16	29751447	29752047	306404	2.5E-06	sparc/osteonectin, cwcv and kazal-like domains proteoglycan (testican) 3
Tubb2a	17	37138997	37140410	498736	1.5E-33	tubulin, beta 2a
Vim	17	87846882	87848477	81818	3.1E-15	vimentin
Pcdhb15	18	30286538	30287543	291646	5.6E-21	protocadherin beta 15
Cdh11	19	2225447	2226332	84407	1.5E-14	cadherin 11
Cdh8	19	5685525	5686520	84408	9.2E-19	cadherin 8
Lrg1	Un/9	25672610	25673300	367455	7.7E-10	leucine-rich alpha-2-glycoprotein 1
Plin5	Un/9	25672610	25673300	501283	7.7E-10	perilipin 5
**Development**						
Dmpk	1	78450272	78451687	308405	8.8E-25	dystrophia myotonica-protein kinase
Six5	1	78450272	78451687	308406	8.8E-25	SIX homeobox 5
Sv2b	1	130887128	130887728	117556	2.3E-14	synaptic vesicle glycoprotein 2b

Gene Symbol	Chr	Start	Stop	Gene ID	min p-value	Gene Title
Usmg5	1	252412922	252413522	171069	1.2E-13	up-regulated during skeletal muscle growth 5 homolog (mouse)
Dkk2	2	229541018	229541911	295445	1.4E-17	dickkopf homolog 2 (Xenopus laevis)
Ntng1	2	205805922	205806522	295382	7.6E-11	netrin G1
Sv2c	2	26554292	26554989	29643	3.1E-08	synaptic vesicle glycoprotein 2c
Cbln4	3	162920485	162921468	228942	9.8E-12	cerebellin 4 precursor
Lhx6	3	15214211	15214897	311901	9.9E-10	LIM homeobox 6
Ntng2	3	8228607	8229412	311836	4.3E-19	netrin G2
SPATA2	3	158640784	158641479	114210	1.6E-26	spermatogenesis associated 2
SMO	4	56653034	56653704	25273	2.2E-12	smoothened homolog (Drosophila)
Dfnb31	5	80377382	80378089	313255	1.6E-09	deafness, autosomal recessive 31
Npc2	6	108814406	108815606	286898	1.4E-36	Niemann-Pick disease, type C2
Trps1	7	87076226	87077124	299897	2.5E-12	trichorhinophalangeal syndrome I homolog (human)
Sox14	8	104731115	104731715	300954	2.1E-09	SRY (sex determining region Y)-box 14
Per1	10	55855898	55856498	287422	4.1E-07	period circadian protein homolog 1 (Drosophila)
Nlgn2	10	56678445	56679248	117096	2.9E-10	neuroligin 2
Hoxb6	10	85032294	85033304	497986	3.9E-26	homeo box B6
HOXB3	10	85078411	85079388	303488	1.8E-20	homeo box B3
Lhx4	13	70704284	70705161	360858	9.0E-12	LIM homeobox 4
Nsg1	14	77932142	77933147	25247	2.2E-10	neuron specific gene family member 1
Irx5	19	15751735	15752415	498918	2.6E-10	iroquois homeobox 5
**Electron Transport**						
Cyp26a1	1	241947986	241948586	154985	1.6E-10	cytochrome P450, family 26, subfamily a, polypeptide 1
**Epigenetics**						
Smarca2	1	230016032	230017142	361745	1.8E-16	SWI/SNF related, matrix associated, actin dependent regulator of chromatin, subfamily a, member 2
Dnmt3a	6	26859491	26860180	444984	3.9E-13	DNA (cytosine-5-)-methyltransferase 3 alpha
H1f0	7	117001533	117002342	24437	4.0E-11	H1 histone family, member 0
Satb2	9	55824749	55825838	501145	1.4E-13	SATB homeobox 2
Tbx2	10	74084425	74085225	303398	6.4E-41	T-box 2
ASMT	12	16815952	16816847	246281	3.6E-20	acetylserotonin O-methyltransferase
Asmtl	12	16824203	16825214	288527	1.1E-10	acetylserotonin O-methyltransferase-like
Kdm2b	12	34672398	34673309	304495	1.9E-19	lysine (K)-specific demethylase 2B
Gadd45g	17	19231639	19232721	291005	7.5E-12	growth arrest and DNA-damage-inducible, gamma

Gene Symbol	Chr	Start	Stop	Gene ID	min p-value	Gene Title
**Golgi Apparatus**						
Ap2a1	1	95403348	95404143	308578	4.4E-11	adaptor-related protein complex 2, alpha 1 subunit
B4galt2	5	138346449	138347345	313536	7.2E-07	UDP-Gal:betaGlcNAc beta 1,4-galactosyltransferase, polypeptide 2
**Growth Factors**						
Fgf15	1	205323456	205324556	170582	2.1E-15	fibroblast growth factor 15
Gdnf	2	57403318	57404513	25453	2.5E-25	glial cell derived neurotrophic factor
Ntf3	4	162504664	162505639	81737	3.3E-13	neurotrophin 3
WNT10B	7	137545980	137547070	315294	1.7E-13	wingless-type MMTV integration site family, member 10B
Sept11	14	16548270	16549359	305227	1.7E-11	septin 11
**Immune Response**						
Siglec10	1	93784998	93785998	292844	4.5E-12	sialic acid binding Ig-like lectin 10
Tmpo	7	28112035	28112635	25359	2.3E-10	thymopoietin
Cd276	8	62361422	62362307	315716	1.8E-09	Cd276 molecule
Lkap	10	822060	822860	170946	6.4E-10	limkain b1
Fcgr2a	13	86913190	86913875	116591	4.1E-10	Fc fragment of IgG, low affinity IIa, receptor (CD32)
RT1-CE7	20	3416588	3417188	368153	3.7E-17	RT1 class I, locus CE7
C4b	20	4103885	4104485	24233	3.9E-11	complement component 4B
RT1-A2	20	5022398	5022998	24974	1.3E-13	RT1 class Ia, locus A2
**Metabolism & Transport**						
Bcat2	1	96038195	96038795	64203	6.3E-07	branched chain aminotransferase 2, mitochondrial
Scd	1	249358774	249359374	83792	2.7E-09	stearoyl-CoA desaturase (delta-9-desaturase)
Fah	1	140875869	140876469	29383	7.8E-09	fumarylacetoacetate hydrolase
Kcnn3	2	181715841	181716441	54263	4.1E-08	potassium intermediate/small conductance calcium-activated channel, subfamily N, member 3
Acoxl	3	115365654	115366254	296138	1.3E-23	acyl-Coenzyme A oxidase-like
Slc32a1	3	149342424	149343525	83612	5.4E-12	solute carrier family 32 (GABA vesicular transporter), member 1
Mat2a	4	105744842	105745527	171347	4.4E-31	methionine adenosyltransferase II, alpha
Magi2	4	11440081	11441061	113970	7.2E-11	membrane associated guanylate kinase, WW and PDZ domain containing 2
Echdc2	5	129265151	129266066	298381	5.8E-10	enoyl Coenzyme A hydratase domain containing 2
Dhrs3	5	163340874	163341672	313689	8.1E-08	dehydrogenase/reductase (SDR family) member 3
Fut9	5	40934221	40934821	84597	1.7E-11	fucosyltransferase 9 (alpha (1,3) fucosyltransferase)
Pusl1	5	172749107	172749987	362681	5.4E-10	pseudouridylate synthase-like 1

Gene Symbol	Chr	Start	Stop	Gene ID	min p-value	Gene Title
Aldh6a1	6	108515786	108516671	81708	8.2E-11	aldehyde dehydrogenase 6 family, member A1
Pygl	6	92340725	92341609	64035	7.8E-10	phosphorylase, glycogen, liver
Syt10	7	128173824	128174714	60567	2.8E-10	synaptotagmin X
Scn3b	8	43230920	43231900	245956	4.3E-08	sodium channel, voltage-gated, type III, beta
Srr	10	62234533	62235133	303306	8.1E-09	serine racemase
KCNJ2	10	100568435	100569338	29712	8.0E-10	potassium inwardly-rectifying channel, subfamily J, member 2
Nmnat2	13	67969800	67970695	289095	2.5E-11	nicotinamide nucleotide adenylyltransferase 2
Tomm40b	13	87111787	87112679	304971	1.0E-11	translocase of outer mitochondrial membrane 40 homolog B (yeast)
Enoph1	14	10859038	10859932	305177	6.9E-41	enolase-phosphatase 1
Dpysl2	15	46412626	46413517	25416	1.9E-09	dihydropyrimidinase-like 2
Kcnn2	18	39559856	39560646	54262	5.7E-10	potassium intermediate/small conductance calcium-activated channel, subfamily N, member 2
Best2	19	24802086	24803166	364973	4.5E-09	bestrophin 2
Prps2	X	47274725	47275523	24689	1.8E-06	phosphoribosyl pyrophosphate synthetase 2
**Proteolysis**						
Ube2h	4	57216643	57217348	296956	1.2E-06	ubiquitin-conjugating enzyme E2H
Metap2	7	31001102	31001702	64370	8.2E-11	methionyl aminopeptidase 2
Prssl1	7	11424819	11425640	408241	6.1E-18	protease, serine-like 1
AMZ2	10	98899341	98900231	360650	2.2E-09	archaelysin family metallopeptidase 2
Mmp17	12	28171455	28172150	288626	1.2E-10	matrix metallopeptidase 17
RGD1560350	20	17488019	17488699	365554	1.2E-09	similar to proteasome subunit iota
Pi16	20	7639255	7640255	294312	2.5E-14	peptidase inhibitor 16
**Receptors & Binding Proteins**						
Abcc6	1	96527328	96528133	81642	4.5E-09	ATP-binding cassette, sub-family C (CFTR/MRP), member 6
Esrra	1	209597756	209598356	293701	1.2E-10	estrogen related receptor, alpha
Pepd	1	87420067	87420667	292808	2.8E-09	peptidase D
Ranbp3l	2	58725173	58726193	294789	4.8E-11	RAN binding protein 3-like
Olr414	3	163798054	163799129	56821	9.3E-16	olfactory receptor 414
Spsb2	4	160931316	160932317	297592	1.0E-12	splA/ryanodine receptor domain and SOCS box containing 2
Olr1070	7	8317229	8317931	366816	3.1E-21	olfactory receptor 1070
Abcc4	15	103052860	103053460	170924	4.0E-14	ATP-binding cassette, sub-family C (CFTR/MRP), member 4
Adra1a	15	46173075	46173997	29412	9.8E-09	adrenergic, alpha-1A-, receptor
Olr1684	20	3497439	3498621	294151	1.1E-10	olfactory receptor 1684
**Signaling**						
Calca	1	172690139	172691119	24241	7.3E-13	calcitonin-related polypeptide alpha
Cnksr3	1	37957713	37958623	308113	4.4E-10	Cnksr family member 3

Gene Symbol	Chr	Start	Stop	Gene ID	min p-value	Gene Title
Gng8	1	77218978	77219578	245986	1.9E-11	guanine nucleotide binding protein (G protein), gamma 8
RAB13	2	182460590	182461570	81756	2.3E-43	RAB13, member RAS oncogene family
ANP32E	2	190715161	190716273	361999	1.4E-15	acidic (leucine-rich) nuclear phosphoprotein 32 family, member E
Egflam	2	56666338	56667335	365691	6.9E-18	EGF-like, fibronectin type III and laminin G domains
Eps8l3	2	203455748	203456642	295361	1.3E-10	EPS8-like 3 (Epidermal growth factor receptor kinase substrate 8-like protein 3)
Notch2	2	192855641	192856541	29492	1.3E-16	Notch homolog 2 (Drosophila)
Dusp15	3	143297017	143298106	362238	5.8E-21	dual specificity phosphatase 15
Acap3	5	172749107	172749987	313772	5.4E-10	ArfGAP with coiled-coil, ankyrin repeat and PH domains 3
LOC683719	5	86273089	86273964	683719	5.6E-11	similar to RAS and EF hand domain containing
Itga7	7	2230364	2231254	81008	4.0E-08	integrin, alpha 7
Palm	7	11424819	11425640	170673	6.1E-18	paralemmin
Shc2	7	11584014	11584614	314612	1.2E-16	SHC (Src homology 2 domain containing) transforming protein 2
Mapk11	7	127442049	127442649	689314	4.7E-13	mitogen-activated protein kinase 11
Shank3	7	127816023	127816717	59312	5.0E-15	SH3 and multiple ankyrin repeat domains 3
Calcoco1	7	141470621	141471436	246047	3.8E-15	calcium binding and coiled coil domain 1
Grasp	7	139968279	139968967	192254	1.8E-07	GRP1 (general receptor for phosphoinositides 1)-associated scaffold protein
Anp32a	8	66488157	66489557	25379	3.8E-09	acidic (leucine-rich) nuclear phosphoprotein 32 family, member A
Snx33	8	60673757	60674357	315696	3.1E-24	sorting nexin 33
Hsp90ab1	9	11032495	11033610	301252	5.1E-10	heat shock protein 90kDa alpha (cytosolic), class B member 1
PLEKHH3	10	90191341	90192426	360634	1.3E-16	pleckstrin homology domain containing, family H (with MyTH4 domain) member 3
Akap17a	12	16824203	16825214	288526	1.1E-10	A kinase (PRKA) anchor protein 17A
Sorbs3	15	50590321	50591217	282843	7.4E-09	sorbin and SH3 domain containing 3
Grk6	17	15237197	15237797	59076	3.7E-13	G protein-coupled receptor kinase 6
Morg1	19	24731570	24733149	288924	4.1E-08	mitogen-activated protein kinase organizer 1
Ppp1r10	20	2984169	2984883	65045	1.2E-29	protein phosphatase 1, regulatory subunit 10
Ppp1r10	20	2987593	2988193	65045	7.7E-09	protein phosphatase 1, regulatory subunit 10
Ptprs	Un	25300105	25300805	25529	9.8E-12	protein tyrosine phosphatase, receptor type, S

Gene Symbol	Chr	Start	Stop	Gene ID	min p-value	Gene Title
Gprasp1	X	1331319	1331919	171407	2.7E-09	G protein-coupled receptor associated sorting protein 1
**Transcription**						
PITX3	1	251356410	251357495	29609	3.5E-36	paired-like homeodomain 3
Ccdc21	5	152927566	152928385	362622	5.7E-18	coiled-coil domain containing 21
Fbxo2	5	165238253	165238963	85273	2.0E-08	F-box protein 2
Ccdc17	5	136879913	136881400	500528	3.1E-14	coiled-coil domain containing 17
NR4A3	5	64719197	64719987	58853	1.2E-13	nuclear receptor subfamily 4, group A, member 3
Tardbp	5	165710469	165711069	298648	2.6E-09	TAR DNA binding protein
Tceb1	5	1975203	1976200	64525	1.2E-10	transcription elongation factor B (SIII), polypeptide 1
RGD1563216	6	108814406	108815606	500694	1.4E-36	similar to HESB like domain containing 1
Batf	6	109793195	109793870	299206	1.6E-08	basic leucine zipper transcription factor, ATF-like
Scx	7	114502670	114504375	680712	6.7E-13	scleraxis
Maff	7	117329082	117330072	366960	6.1E-11	v-maf musculoaponeurotic fibrosarcoma oncogene homolog F (avian)
Bcl9l	8	47449801	47450705	300673	1.4E-14	B-cell CLL/lymphoma 9-like
Klhdc3	9	10052217	10053619	363192	1.4E-07	kelch domain containing 3
Foxp4	9	8507797	8509094	363185	7.5E-12	forkhead box P4
Ubtf	10	91376259	91377139	25574	1.8E-09	upstream binding transcription factor, RNA polymerase I
Scand3	12	27861394	27861994	288622	4.7E-09	SCAN domain containing 3
Higd2al1	12	1366187	1366787	688606	7.1E-07	HIG1 hypoxia inducible domain family, member 2A-like 1
Atf3	13	107225329	107225929	25389	1.9E-07	activating transcription factor 3
Ndrg2	15	27346660	27347666	171114	1.1E-25	N-myc downstream regulated gene 2
Zic5	15	107598237	107599148	361095	2.6E-17	Zic family member 5 (odd-paired homolog, Drosophila)
Ing1	16	82793987	82794978	306626	1.1E-07	inhibitor of growth family, member 1
Gata6	18	2502698	2503598	29300	6.2E-09	GATA binding protein 6
Klhl14	18	13281385	13282422	364823	1.2E-09	kelch-like 14 (Drosophila)
Fbxl8	19	35084702	35085522	498941	1.1E-12	F-box and leucine-rich repeat protein 8
Armcx2	X	122265349	122266237	367903	7.8E-33	armadillo repeat containing, X-linked 2
**Translation & Protein Modification**						
Cstf3	3	89919001	89919799	362178	1.3E-13	cleavage stimulation factor, 3' pre-RNA, subunit 3
Eef2	7	10021587	10022487	29565	1.0E-14	eukaryotic translation elongation factor 2
Rnasek	10	57077243	57078332	287453	1.3E-08	ribonuclease, RNase K
Igf2bp1	10	84714200	84715211	303477	8.0E-10	insulin-like growth factor 2 mRNA binding protein 1
Npm1	10	18062193	18062873	25498	7.1E-10	nucleophosmin (nucleolar phosphoprotein B23, numatrin)

Gene Symbol	Chr	Start	Stop	Gene ID	min p-value	Gene Title
Eif4g1	11	82470413	82471209	287986	4.8E-10	eukaryotic translation initiation factor 4 gamma, 1
Hnrpdl	14	10859038	10859932	305178	6.9E-41	heterogeneous nuclear ribonucleoprotein D-like
Mrps18b	20	2984169	2984883	294230	1.2E-29	mitochondrial ribosomal protein S18B
Mrps18b	20	2987593	2988193	294230	7.7E-09	mitochondrial ribosomal protein S18B
Rpl36	Un	25163917	25164597	58927	1.9E-14	ribosomal protein L36
**Miscellaneous & Unknown**						
Giyd2	1	185849246	185850026	293489	1.4E-12	GIY-YIG domain containing 2
Tsku	1	155621758	155622558	308843	2.8E-21	tsukushin
NSCAN chr1.2257.a	1	220861164	220861964		3.8E-08	
NSCAN pred chr1.948.a	1	101299220	101299820		4.1E-18	
RGD1308958	5	70448054	70449168	298020	7.5E-10	similar to chromosome 9 open reading frame 5
Birc6	6	20847680	20848580	313876	2.0E-09	baculoviral IAP repeat-containing 6
NSCAN pred chr6.009.a	6	1695151	1695930		8.6E-18	
NSCAN pred chr6.1025.a	6	143691197	143692192		4.8E-16	
RGD1308134	10	57077243	57078332	287452	1.3E-08	similar to RIKEN cDNA 1110020A23
Tmem119	12	43859955	43860555	304581	2.8E-11	transmembrane protein 119
NSCAN pred chr12.007.a	12	1019708	1020388		1.6E-08	
RGD1307161	13	99339499	99340519	305031	1.4E-19	similar to 0610010K06Rik protein
Tmem33	14	43516113	43517008	59303	2.9E-12	transmembrane protein 33
NSCAN pred chr14.127.a	14	14702118	14702812		1.2E-08	
NSCAN pred chr18.057.a	18	6993296	6994296		5.4E-08	
NSCAN chr20.066.a	20	3442365	3443350		1.3E-33	
NSCAN pred chrX.212.a	X	28736330	28736930		1.1E-08	
EST's						
TL0AEA6YH06 mRNA seq	18	45009969	45010947		1.4E-06	
TL0AEA9YC13 mRNA seq	18	72291368	72291968		5.0E-07	
RGD1564058	19	24731570	24733149	288925	4.1E-08	similar to cDNA sequence BC056474
