# Supplementary material for: Plastics Derived Endocrine Disruptors (BPA, DEHP and DBP) Induce Epigenetic Transgenerational Inheritance of Obesity, Reproductive Disease and Sperm Epimutations
Source: PLoS One. 2013 Jan 24;8(1):e55387. doi: 10.1371/journal.pone.0055387 (PMC3554682; doi:10.1371/journal.pone.0055387)
Supplement: Table S5 — The F3 generation plastic lineage sperm DMR associated genes correlation to KEGG pathways. The pathway name, the number of DMR genes, and total number of genes in the pathway are listed. (PDF) [file pone.0055387.s007.pdf]

Supplemental Table S5.

Top KEGG Pathways Enriched with Plastics Sperm DMR Group Gene Lists

Pathway Name	# Genes affected	# Genes in Pathway
Endocytosis	5	231
Cell adhesion molecules (CAMs)	5	151
Pathways in cancer	5	321
MAPK signaling pathway	4	253
Ubiquitin mediated proteolysis	4	127
Phagosome	4	191
HTLV-I infection	4	296
Cysteine and methionine metabolism	3	36
Chemokine signaling pathway	3	178
Focal adhesion	3	187
ECM-receptor interaction	3	74
Tight junction	3	128
Antigen processing and presentation	3	100
Neurotrophin signaling pathway	3	129
Olfactory transduction	3	732
Regulation of actin cytoskeleton	3	204
Herpes simplex infection	3	207
Systemic lupus erythematosus	3	114
Hypertrophic cardiomyopathy (HCM)	3	85
Dilated cardiomyopathy	3	94
Viral myocarditis	3	109
